# *LINC00589*-dominated ceRNA networks regulate multiple chemoresistance and cancer stem cell-like properties in HER2^+^ breast cancer

**DOI:** 10.1038/s41523-022-00484-0

**Published:** 2022-10-29

**Authors:** Wendong Bai, Hongyan Peng, Jiarui Zhang, Yongmei Zhao, Zhijun Li, Xuelian Feng, Jiang Zhang, Fei Liang, Li Wang, Nan Zhang, Yize Li, Huayu Zhu, Qiuhe Ji

**Affiliations:** 1 Endocrinology Research Center, Department of Endocrinology and Metabolism, Xijing Hospital, Fourth Military Medical University, 710032 Xi’an, China; 2 Department of Hematology, Xinjiang Command General Hospital of Chinese People’s Liberation Army, 830000 Urumqi, China; 3 Department of Internal Medicine, 63650 Military Hospital, 830000 Urumqi, China; 4 Department of Pathology, Tangdu Hospital, Fourth Military Medical University, 710038 Xi’an, China; 5 Department of Clinical Oncology, Xijing Hospital, Fourth Military Medical University, 710032 Xi’an, China; 6 Department of Burns and Cutaneous Surgery, Xijing Hospital, Fourth Military Medical University, 710032 Xi’an, China

**Keywords:** Breast cancer, Non-coding RNAs, Cancer therapeutic resistance, Prognostic markers

## Abstract

Resistance to human epidermal growth factor receptor 2 (HER2)-targeted therapy (trastuzumab), cancer stem cell (CSC)-like properties and multiple chemoresistance often concur and intersect in breast cancer, but molecular links that may serve as effective therapeutic targets remain largely unknown. Here, we identified the long noncoding RNA, *LINC00589* as a key regulatory node for concurrent intervention of these processes in breast cancer cells in vitro and in vivo. We demonstrated that the expression of *LINC00589* is clinically valuable as an independent prognostic factor for discriminating trastuzumab responders. Mechanistically, *LINC00589* serves as a ceRNA platform that simultaneously sponges *miR-100* and *miR-452* and relieves their repression of tumor suppressors, including discs large homolog 5 (*DLG5*) and PR/SET domain 16 (*PRDM16*, a transcription suppressor of *mucin4*), thereby exerting multiple cancer inhibitory functions and counteracting drug resistance. Collectively, our results disclose two *LINC00589*-initiated ceRNA networks, the *LINC00589*-*miR-100*-*DLG5* and *LINC00589*-*miR-452*-*PRDM16*- *mucin4* axes, which regulate trastuzumab resistance, CSC-like properties and multiple chemoresistance of breast cancer, thus providing potential diagnostic and prognostic markers and therapeutic targets for HER2-positive breast cancer.

## Introduction

Breast cancer is the most prevalent malignancy, with high worldwide mortality in women. Overexpression of human epidermal growth factor receptor 2 (HER2) occurs in 25–30% of breast cancers and is associated with poor prognosis^1,2^. Though HER2-targeted therapy, such as trastuzumab, improves survival dramatically and is the most highly recommended treatment for HER2-positive patients with early-stage or metastatic breast cancer, high rates of inherent or acquired trastuzumab resistance pose a major obstacle^3,4^. In addition, emerging evidence suggest that trastuzumab resistance is closely associated with epithelial–mesenchymal transition (EMT), multiple drug resistance (MDR), and cancer stem cell (CSC)-like properties, which make it more complex to treat trastuzumab-resistant breast cancers^5^. However, key regulatory nodes that are concurrently involved in trastuzumab resistance, MDR, and CSC properties have yet to be uncovered. It is urgent to identify molecular links that concurrently regulate these processes as an approach to developing more effective therapeutic targets.

Noncoding RNAs (ncRNAs) compose the large majority (~98%) of human transcriptome and participate as key players in diverse biological processes^6^. Long noncoding RNAs (lncRNAs) are a class of ncRNAs that are longer than 200 nucleotides and have limited or no protein-coding capacity^7^. LncRNAs, in conjunction with microRNAs or other signaling partners, regulate complex networks, and recent evidence is emerging for key roles of lncRNAs as regulators and potential targets in breast cancer. For example, LncRNA CARMN, acting via the miR143-3p host gene, counteracts cisplatin resistance in triple-negative breast cancer and is associated with positive prognosis^8^. LncRNA CCAT1 interacts with miR-204/211, miR-148a/152, and Annexin A2, and consequently promotes breast cancer stem cell function by activating WNT/β-catenin signaling^9^. Our previous reports also suggest that ncRNAs, including lncRNA UCA1, miR-200c, miR-221, and miR-375, reverse trastuzumab resistance of HER2-positive breast cancer^1,3,10,11^. Therefore, extensive investigation of lncRNAs is important for the development of novel diagnostic and therapeutic targets in breast cancer.

Long intergenic non-protein-coding RNA 589 (*LINC00589*, NCBI gene ID: 619351), also known as TSLNC8, is located on Chromosome 8p12 and has been validated as a non-protein-coding RNA. In hepatocellular carcinoma, non-small cell lung cancer, and glioma, *LINC00589* inhibits proliferation, invasion, and metastasis^12^. On the other hand, in pancreatic cancer, *LINC00589* serves as an oncogene by stabilizing CTNNB1^13^. These results imply that *LINC00589* is important in cancer progression but that its functions vary among different cancer types^14,15^. Nevertheless, the role of *LINC00589* in breast cancer has not been elucidated.

In this study, we evaluate the expression of *LINC00589* in trastuzumab-resistant breast cancer tissues and cell lines, and analyze its association with patient prognosis. We also perform gain- and loss-of-function experiments to explore the biological roles of *LINC00589* in trastuzumab resistance, MDR, and CSC properties in vitro and in vivo. Furthermore, we investigate the molecular mechanisms whereby *LINC00589* exerts its diverse functions in HER2 breast cancer. Our data show that *LINC00589* concurrently modulates trastuzumab resistance, MDR and CSC-like properties of HER2 -positive breast cancer. Further mechanistic investigations reveal that *LINC00589* serves as a competing endogenous RNA (ceRNA) to regulate *DLG5* and *PRDM16* expression through binding *miR-100* and *miR-452*. Thus, *LINC00589* is a key node for simultaneously controlling trastuzumab resistance, MDR, and CSC-like properties in breast cancer with potential therapeutic value.

## Results

### *LINC00589* expression is decreased in trastuzumab-resistant breast cancer and is correlated with the prognosis of HER2-positive breast cancer

**Fig. 1:**
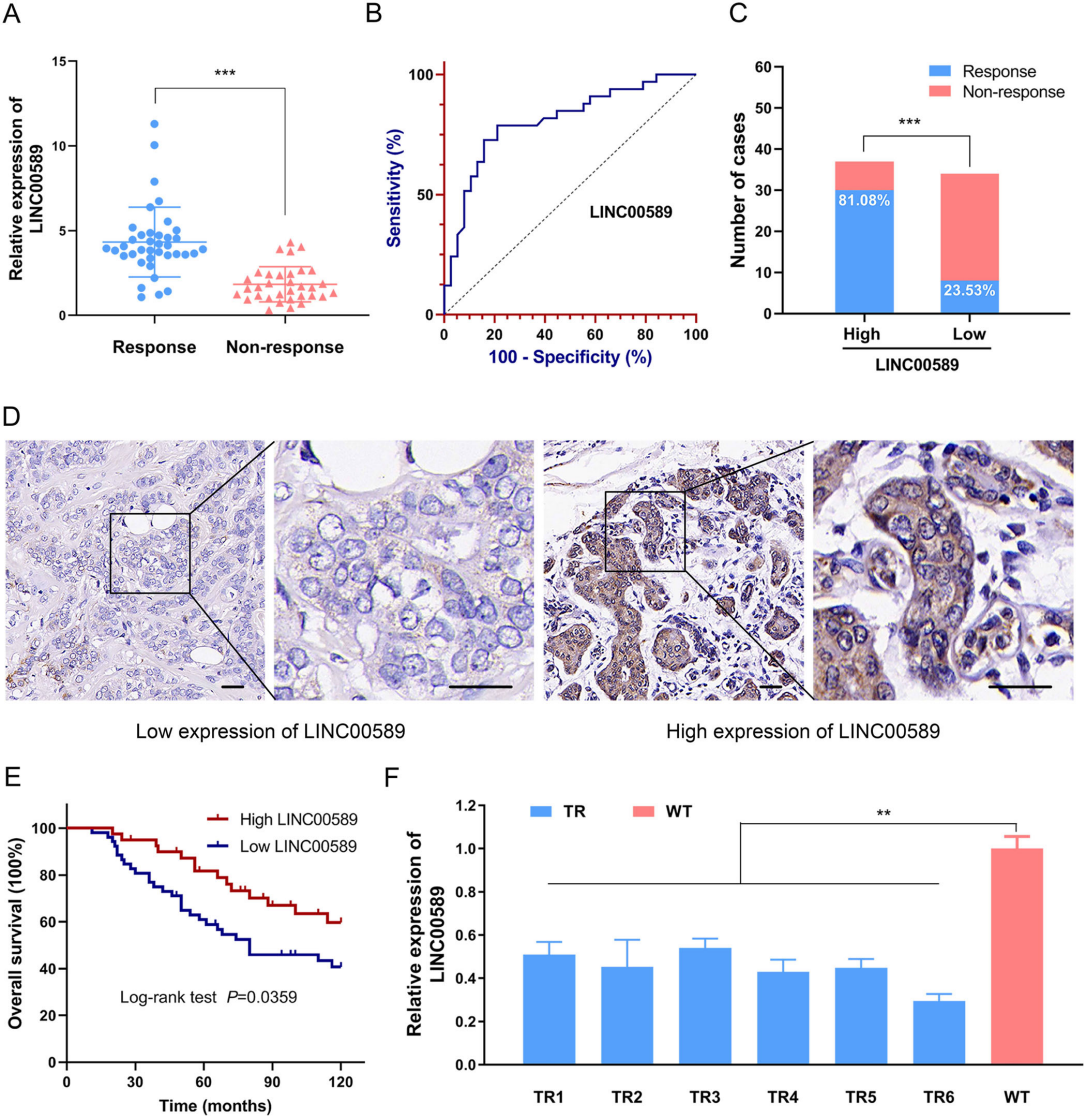
*LINC00589* expression is associated with response to trastuzumab in HER2-positive breast cancer. **A** mRNA expression of *LINC00589* in trastuzumab-responding (*N* = 38) and non-responding (*N* = 33) breast cancer patients was detected by qRT-PCR. Data were analyzed by a two-tailed *t* test. **B** ROC curve for *LINC00589* expression to differentiate responding patients from non-responding patients. **C** The rate of trastuzumab-response patients was significantly higher in the high *LINC00589* expression groups than in the low expression group. **D** In situ hybridization (ISH) staining of high and low expression of *LINC00589* in formalin-fixed and paraffin-embedded tissues from HER2-positive breast cancer patients with trastuzumab treatment. Scale bar 50 μm. **E** Kaplan–Meier’s correlation analyses between *LINC00589* expression and overall survival of patients (low, *N* = 43; high, *N* = 49). **F** The expression of *LINC00589* in TR clone cells and WT cells was evaluated by qRT-PCR. Assays were conducted in triplicate. Data are shown as the means ± SD; data were analyzed by ANOVA and two-tailed *t* test. **P < 0.01, ***P < 0.001.

The long noncoding RNA *LINC00589*, is located on Chromosome 8p12 and contains four exons (Supplementary Fig. 1a). Its full-length 1413 bp nucleotides (Supplementary Fig. 1b) and the secondary structure (Supplementary Fig. 1c) were shown. Although *LINC00589* has been reported to suppress cell proliferation in hepatocellular carcinoma and non-small cell lung cancer^13,16^, its biological roles are largely unknown. Especially, its roles in drug resistance to breast cancer have not been investigated prior to this study. Therefore, to uncover the potential functions of *LINC00589* in trastuzumab-resistant breast cancer, we obtained biopsies from 71 HER2-positive breast cancer patients who received trastuzumab treatment. Based on the immuno-related response evaluation criteria in solid tumors^17^, the patients were divided into two groups: the responding group (CR + PR, 38 cases) and the non-responding group (SD + PD, 33 cases). qRT-PCR analysis of biopsies revealed a dramatically lower expression of *LINC00589* in the trastuzumab non-responding group than in the responding group (Fig. 1A). To investigate the potential predictive value of *LINC00589* expression, we established a ROC curve to differentiate the responding patients from the non-responding patients. The area under the curve (AUC), diagnostic sensitivity, and specificity reached 0.808, 78.8% and 80.0%, respectively, with the established cut-offs (2.785) (Fig. 1B). For further verification, we divided the samples into high or low *LINC00589* expression groups according to the cutoff value, and the proportion of responding patients was significantly higher in the high *LINC00589* expression group (81.08%) than in the low *LINC00589* expression group (23.53%) (Fig. 1C). These results suggest that *LINC00589* may serve as a diagnostic marker for trastuzumab-responding patients.

To further evaluate the prognostic value of *LINC00589* expression, we obtained formalin-fixed and parrffin-embedded (FFPE) samples from an independent cohort of 92 trastuzumab-treated HER2-positive breast cancer patients with available clinical data. High and low expression of *LINC00589* were determined by ISH as represented in Fig. 1D. The results suggest that there is no obvious correlation between *LINC00589* expression and age, menopausal status, histologic grade, lymph node status, ER status or PR status in HER2-positive breast cancer patient tissues; however, *LINC00589* expression was significantly correlated with TNM stage (Table 1). Furthermore, Kaplan–Meier analysis indicated that HER2-positive breast cancer patients with high *LINC00589* expression had a better overall survival than those with low *LINC00589* expression (Fig. 1E). In addition, multivariate Cox regression analysis revealed that *LINC00589* expression and lymph node status provided independent prognostic factors for overall survival in the HER2-positive breast cancer patients (Table 2). To provide additional support for the correlation of *LINC00589* expression with trastuzumab resistance, we treated SKBR3 breast cancer cells with 5 μg/mL trastuzumab for 6 months, as previously described^1,3,10^, and obtained 6 trastuzumab-resistant (TR) clones. The IC50 for 6 TR clones was much upper than WT clone, and IC50 value of 6# TR clones was 24 μg/ml (Supplementary Fig. 2). *LINC00589* expression was dramatically lower in all the TR cell clones than in the wild-type cells (WT) cells (Fig. 1F). The 6# TR cell clone that expresses the lowest *LINC00589* was selected for further investigation the role of *LINC00589* in trastuzumab resistance. Compared with WT cells, the TR cells showed more resistant to trastuzumab treatment, as evidenced by elevated cell viability and IC50 (Supplementary Fig. 3). Altogether, our data indicate that *LINC00589* expression is downregulated in trastuzumab-resistant breast cancer and correlates with patient survival, suggesting that *LINC00589* may be a valuable diagnostic marker for discriminating trastuzumab responders and a prognostic marker for predicting the survival of HER2-positive breast cancer patients.

**Table 1:** Association of LINC00589 expression with clinicopathological features in trastuzumab-treated HER2-positive breast cancer patients.

Characteristics	Patients with FFPE tissue (*n* = 92)	LINC00589 expression		*χ*^2^	*P*
		Low	High		
Age				0.076	0.783
<50	29	17	12		
≥50	63	35	28		
Menopausal status				0.050	0.824
Pre		22	16		
Post		30	24		
Histological grade				1.172	0.279
Grade I–II		38	25		
Grade III		14	15		
TNM stage				4.284	0.038^*^
Tu I–II		36	35		
Tu III		16	5		
Lymph node status				0.086	0.769
Negative		25	18		
Positive		27	22		
ER status				0.282	0.596
Negative		10	6		
Positive		42	34		
PR status				0.028	0.868
Negative		23	17		
Positive		29	23		

*FFPE* formalin-fixed and paraffin-embedded, lymph node status: negative, number of nodal metastases ≤3; positive, number of nodal metastases >3, *ER* estrogen receptor, *PR* progesterone receptor.

The *χ*^2^ test was used to compare percentages or the association between *LINC00589* and clinicopathological parameters. *P < 0.05

**Table 2:** Multivariate analysis for breast cancer patients with trastuzumab treatment.

Characteristics	Overall survival	
	*P*	HR (95% CI)
Age	0.840	1.071 (0.550–2.084)
Menopausal status	0.135	0.609 (0.318–1.166)
Histologic grade	0.440	0.760 (0.379–1.523)
TNM stage	0.607	1.218 (0.574–2.585)
Lymph node status	<0.001	3.885 (1.880–8.026)
ER status	0.925	1.045 (0.420–2.601)
PR status	0.531	0.812 (0.424–1.558)
*LINC00589* expression	0.021	0.453 (0.231–0.887)

*HR* hazard ratio, lymph node status: negative, number of nodal metastases ≤3; positive, number of nodal metastases >3, *ER* estrogen receptor, *PR* progesterone receptor, *CI* confidence interval.

Data were analyzed by cox regression.

### *LINC00589* counteracts trastuzumab resistance in HER2-positive breast cancer

**Fig. 2:**
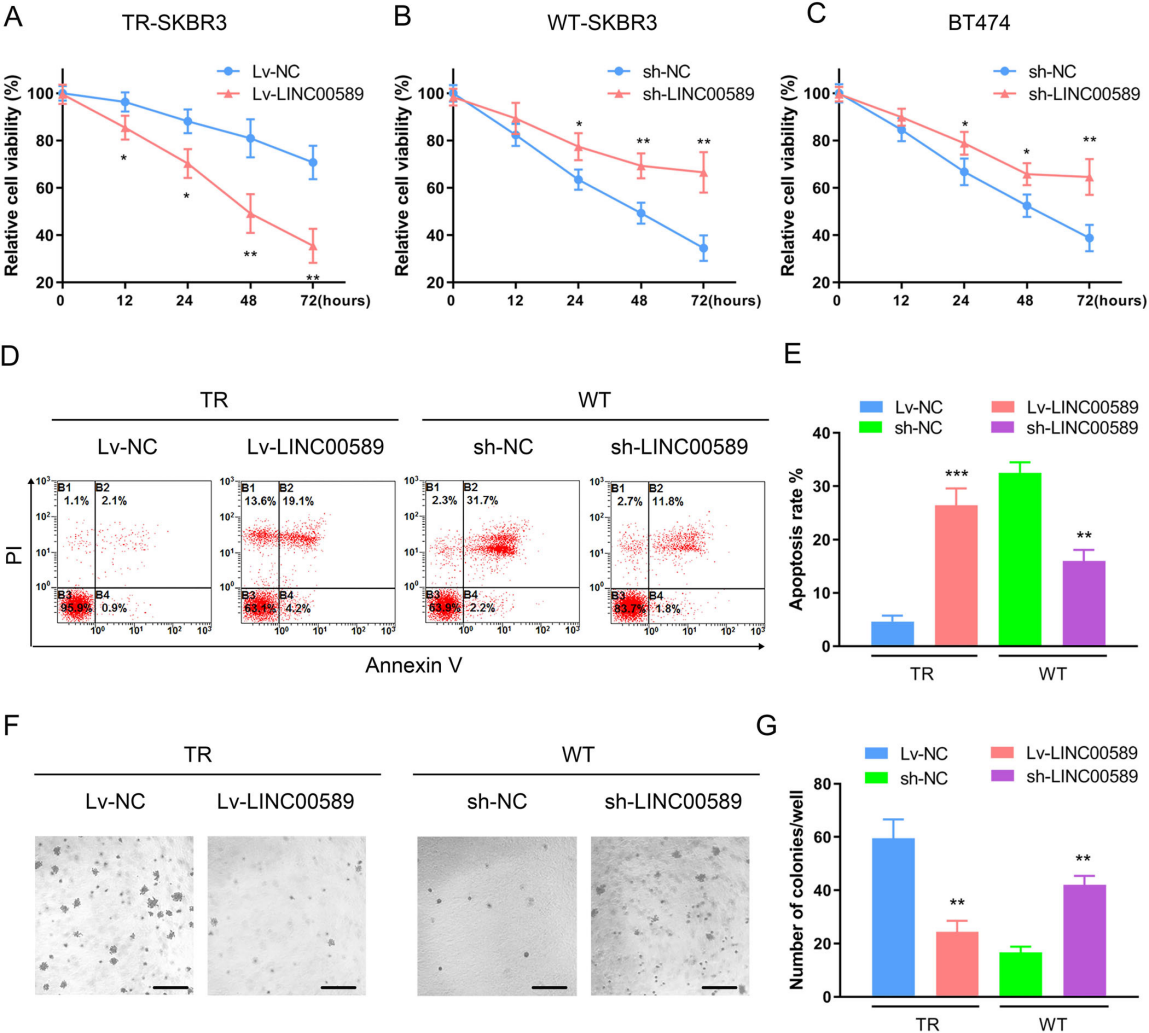
*LINC00589* promotes the sensitivity of breast cancer cells to trastuzumab and inhibits anchorage-independent growth. **A**–**C** TR SKBR3 cells were infected with NC or *LINC00589*-overexpression lentivirus and treated with 25 μg/ml trastuzumab. WT SKBR3 and BT474 cells were infected with sh-NC or sh-*LINC00589* lentivirus and treated with 5 μg/ml trastuzumab treatment. The relative cell viabilities were determined by CCK-8 assays at the indicated times. **D**, **E** TR and WT SKBR3 cells were infected with overexpression or shRNA lentiviruses and then were treated with 25 μg/ml trastuzumab or 5 μg/ml trastuzumab for 48 h, followed by FITC-conjugated annexin V and PE-labeled PI staining. The apoptosis rate was analyzed by flow cytometry. **F**, **G** TR or WT SKBR3 cells were cultured in soft agar for 21 days after trastuzumab treatment and then subjected to crystal violet staining. The clones were observed, and the number of clones for each sample was calculated. Scale bar 100 μm. Data are represented as the mean ± SD of three replicates or are representative of three independent experiments. Two-way ANOVA were used to analyze the data in (**A**–**C**), and two-tailed *t* test was used to analyze the data in (**E**, **G**). *P < 0.05, **P < 0.01, and ***P < 0.001 versus negative control (NC).

To determine the functional role of *LINC00589* in trastuzumab resistance of HER2-positive breast cancer cells, we constructed lentiviruses that overexpress or silence *LINC00589* (Supplementary Fig. 4a, b). WT and TR SKBR3 and HER2-overexpressing BT474 cells were infected with Lv-NC or Lv-*LINC00589* lncRNA expression vector, or sh-NC or sh-*LINC00589* lentivirus. CCK-8 assays revealed that overexpression of *LINC00589* decreased the cell viability of all the six TR SKBR3 cells (Fig. 2A and Supplementary Fig. 5), while knockdown of *LINC00589* increased cell viability in WT SKBR3 and BT474 breast cancer cells, which was verified under increasing doses (Supplementary Fig. 6a–c) or times (Fig. 2B, C) of trastuzumab treatment. In addition, the apoptosis rate of TR cells was increased by *LINC00589* overexpression after trastuzumab treatment, while the apoptosis rate of WT cells was decreased by *LINC00589* silencing (Fig. 2D, E). We also investigated whether *LINC00589* regulates the anchorage-independent growth of HER2-positive breast cancer cells. The data showed that *LINC00589* upregulation suppressed the number of soft agar colonies formed in TR cells, while knockdown of *LINC00589* increased the number of soft agar colonies formed in WT cells (Fig. 2F, G). Collectively, these findings indicate that *LINC00589* re-sensitizes resistant breast cancer cells to trastuzumab.

### *LINC00589* reverses cancer stem cell-like properties and multiple chemoresistance in trastuzumab-resistant HER2-positive breast cancer

**Fig. 3:**
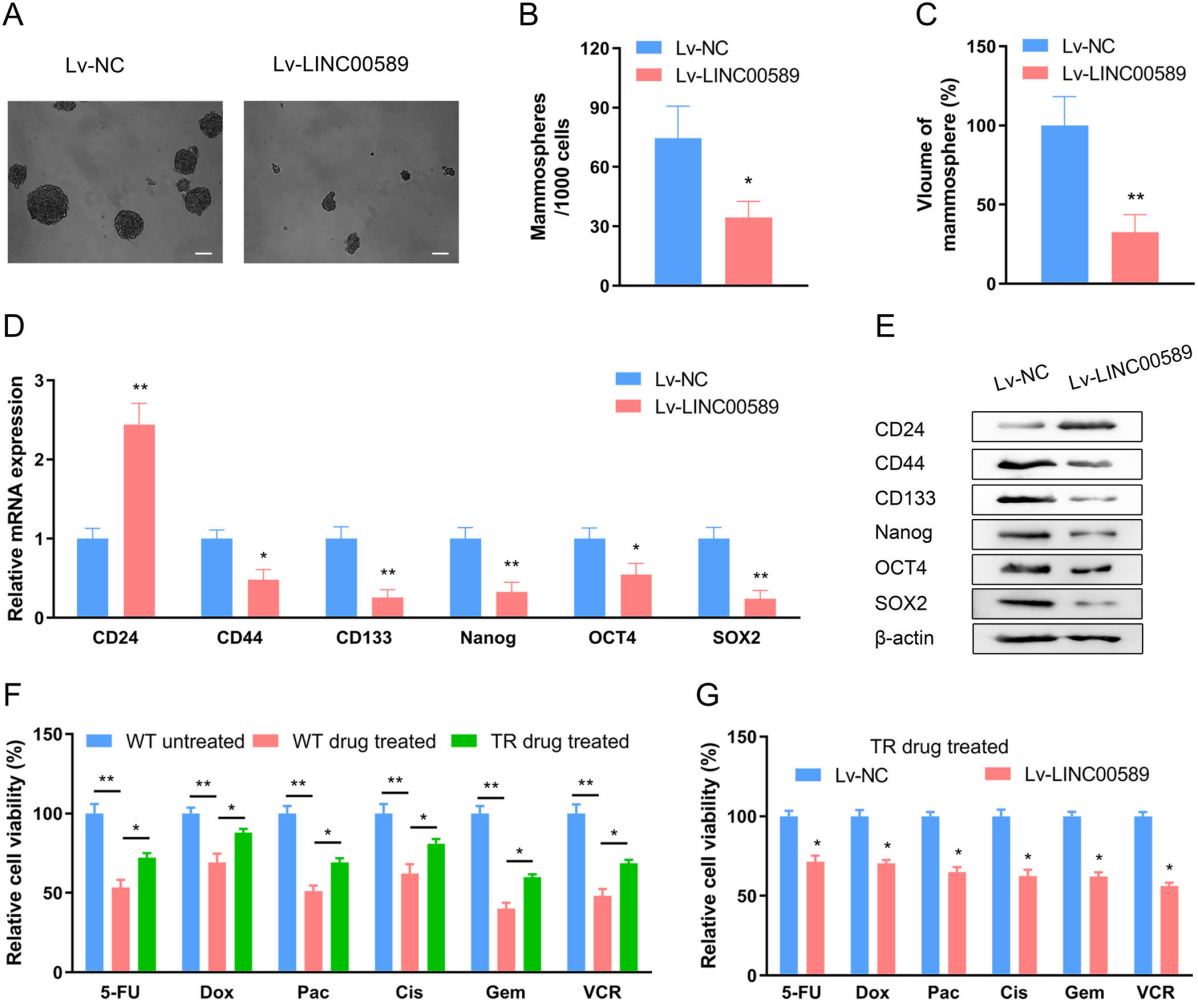
*LINC00589* expression reverses cancer stem cell-like properties and multiple chemoresistance of HER2-positive breast cancer. **A** Representative image of mammosphere formation in NC- and *LINC00589*- overexpressing TR breast cancer cells. Scale bar 100 μm. **B**, **C** Quantification of the mammosphere number (**B**) and volume (**C**). Data are shown as the mean ± SD of five random high-power fields (HPF), and were analyzed by two-tailed *t* test. *P < 0.05, and **P < 0.01. **D**, **E** TR cells were infected with NC lentivirus or Lv-LINC0089. mRNA and protein levels of molecular markers of breast cancer CSC-like properties were determined by qRT-PCR assay (**D**) and western blotting assay (**E**). Data are shown as the mean ± SD and were analyzed by Student’s unpaired two-tailed *t* test. *P < 0.05, and **P < 0.01. **F**, **G** WT cells, TR cells, and TR cells infected with NC- or *LINC00589*-overexpressing lentivirus were treated with 5-FU, doxorubicin (Dox), paclitaxel (Pac), cisplatin (Cis), gemcitabine (Gem), or vincristine (VCR) for 48 h. The relative cell viability was tested by CCK-8 assay and normalized to non-treated cells. Data are shown as mean ± SD and were analyzed by two-tailed *t* test. *P < 0.05, and **P < 0.01 versus negative control (NC).

Based on increasing evidence that trastuzumab-resistant breast cancer cells exhibit CSC-like properties^18^, we sought to determine whether *LINC00589* is associated with stemness and multiple chemoresistance in breast cancer cells. The ability to form mammospheres in ultra-low–attaching culture conditions is a common characteristic of CSC-like cells. As shown in Fig. 3, The average number and volumes of the spheres derived from the *LINC00589*-overexpressed trastuzumab-resistant cells were lower than those derived from control cells (Fig. 3A–C). We also examined the expression status of CD24, CD44, CD133, Nanog, OCT4, and SOX2, which have been extensively used as molecular markers for breast CSCs^19^. When *LINC00589* was overexpressed in TR cells, CD24 (a negative marker of CSC) was upregulated, and CD44, CD133, Nanog, OCT4, and SOX2 (positive markers of CSC) were downregulated, which was demonstrated at both the mRNA and protein levels (Fig. 3D, E). These results indicated that *LINC00589* was an important regulator of CSC-like properties in breast cancer.

Given that CSC-like properties are thought to constitute a leading cause for multiple drug resistance of various cancers^20^, we hypothesized that trastuzumab-resistant breast cancer cells might acquire multiple chemoresistance. Thus, we used several first-line chemotherapeutic drugs for breast cancer, including 5-FU, doxorubicin (Dox), paclitaxel (Pac), cisplatin (Cis), gemcitabine (Gem), and vincristine (VCR), to examine the multiple chemoresistance of TR cells. Compared to WT cells, TR breast cancer cells displayed less sensitivity to each of these drugs (Fig. 3F). However, overexpression of *LINC00589* remarkably re-sensitized the TR cells to all of these drugs (Fig. 3G). Consistently, we determined HER2 expression by qRT-PCR and western blot assays (Supplementary Fig. 7a–c), and observed decreased HER2 expression in TR cell lines. Meanwhile, *LINC00589* overexpression could not change HER2 expression at both mRNA and protein levels in trastuzumab-resistant breast cancer cells (Supplementary Fig. 7d–f). This suggested that *LINC00589* might exerted multiple functions through a HER2-independent mechanism in HER2-positive breast cancer. Consistently with the in vitro experiment, we also observed no correlation between *LINC00589* and HER2 expression in patients’ tissues in clinical samples (Supplementary Fig. 7g). Taken together, the above findings suggest that *LINC00589* decreases CSC-like properties and reverses the resistance of TR cells to multiple chemotherapeutic agents.

### *LINC00589* functions as a ceRNA and sponges *miR-100* and *miR-452* in breast cancer cells

**Fig. 4:**
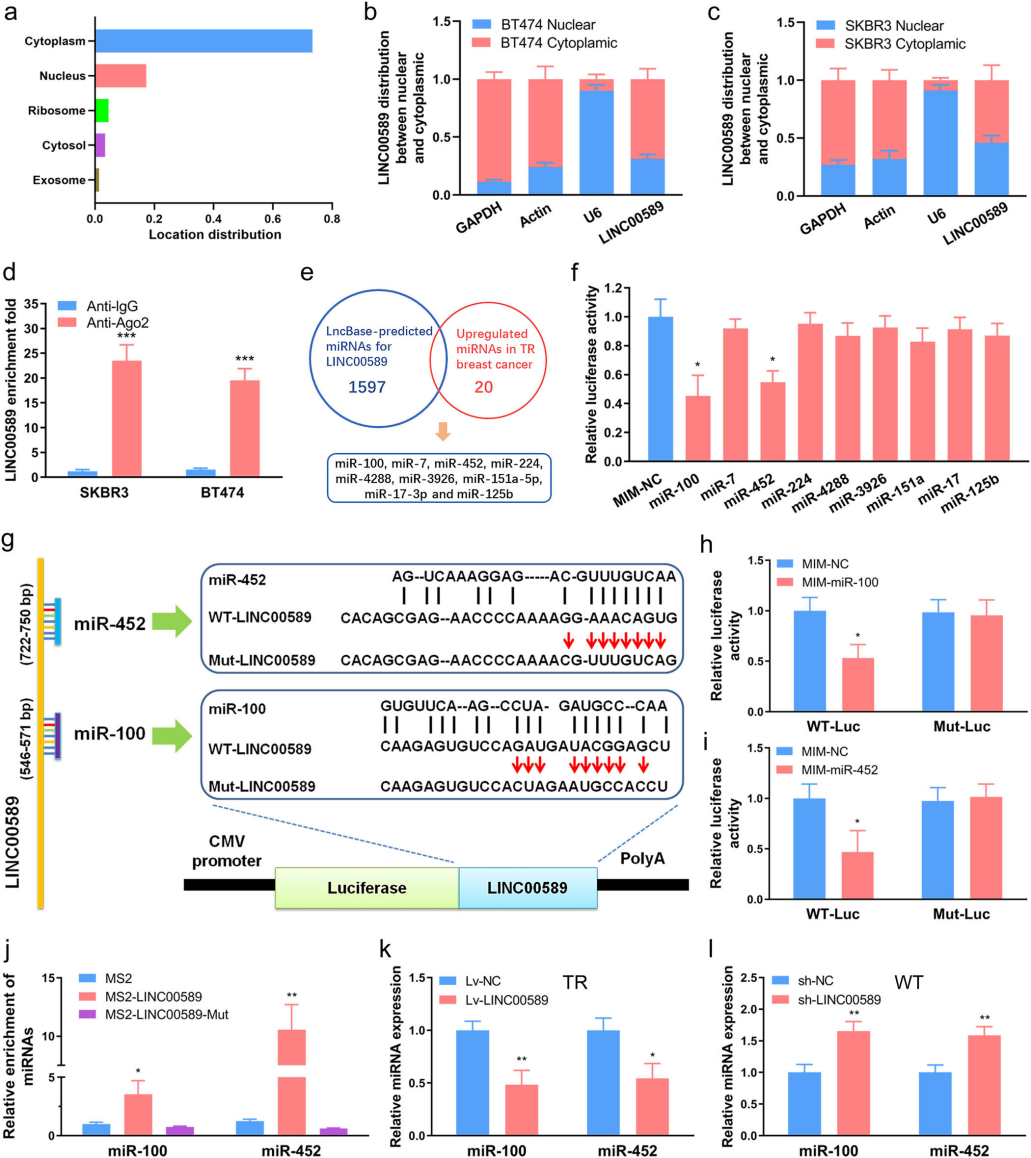
*LINC00589* sponges *miR-100* and *miR-452*. **a** *LINC00589* localization was predicted using the lncRNA subcellular localization predictor lncLocator. **b**, **c** *LINC00589* localization was confirmed by subcellular fractionation assays in BT474 and SKBR3 cells. Nuclear control: U6; cytosolic control: GAPDH. **d** RNA immunoprecipitation experiments were performed in BT474 and SKBR3 cells, and the coprecipitated RNA was used to quantify *LINC00589* expression by qRT-PCR. **e** Potential miRNAs binding to *LINC00589* via miRNA-*LINC00589* interaction were predicted by lncBase. Then the predicted miRNAs and the upregulated miRNAs in TR cells from GEO dataset (GSE47011) were overlapped. Nine candidate miRNAs were obtained for further investigation. **f** Luc-*LINC00589* vector was co-transfected with the NC mimic (MIM) or the nine miRNAs MIM into TR cells for 48 h, followed by luciferase reporter assay. **g** Predicted *miR-100* and *miR-452* binding sites on *LINC00589* and the mutations for luciferase reporter assay are shown. **h**, **i** Luciferase reporter plasmids containing wild type (WT) or mutant (Mut) *LINC00589* were co-transfected with NC MIM, *miR-100* MIM, or *miR-452* MIM into TR cells for 48 h. Subsequently, the luciferase activity was evaluated. **j** MS2, MS2-*LINC00589* or MS2-*LINC00589* harboring mutations (Mut) in the *miR-100* or *miR-452* binding sites were transfected into WT cells for 48 h. Then, total RNA was incubated with MBP-MCP-conjugated amylose resin, and the immunoprecipitated RNA was subjected to qRT-PCR. **k**, **l** Expression of *miR-100* and *miR-452* in TR cells infected with Lv-NC or Lv-*LINC00589* in WT cells infected with sh-NC or sh-*LINC00589* was measured by qRT-PCR assay. U6 was used as an internal control. Data are shown as mean ± SD; two-tailed *t* test was used to analyze the data in (**d**, **f**, **h**, **I**, **j**, **k**, and **l**). *P < 0.05, **P < 0.01 and ***P < 0.001 versus negative control (NC).

Functional roles of lncRNAs are associated with their cellular localization. To distinguish potential molecular mechanisms whereby *LINC00589* exerts its multiple functions in HER2-positive breast cancer, we determined its cellular location. The online lncLocator software predicted that *LINC00589* is mainly enriched in the cytoplasm (Fig. 4a). Consistently, subcellular fractionation assays revealed that *LINC00589* is mostly distributed in the cytoplasm in both BT474 and SKBR3 cells (Fig. 4b, c). These results raise the possibility that *LINC00589* might regulate target protein expression at the post-transcriptional level. As ceRNA mechanism is an important mode for cytoplasmic lncRNA-mediated post-transcriptional regulation^21^, we hypothesized that *LINC00589* may competitively sponge miRNAs. To test this hypothesis, we performed an immunoprecipitation assay for Ago2, an important protein component of the RNA-induced silencing complex. The results demonstrate that *LINC00589* bind to with Ago2 and was involved in the miRNA-mediated repression of mRNA (Fig. 4d). To further investigate the miRNAs that may be sponged by *LINC00589*, we used lncBase and obtained 1597 potential binding miRNAs for *LINC00589*. As we have previously performed a microarray between WT and TR SKBR3 cells (GSE47011)^1^, we selected the most highly upregulated miRNAs (fold change >4.0) and evaluated overlap with lncBase-predicted miRNAs, which yielded 9 candidate miRNAs, including *miR-100* (*miR-100-5p*)*, miR-7* (*miR-7-5p*)*, miR-452* (*miR-452-5p*)*, miR-224* (*miR-224-5p*)*, miR-4288, miR-3926, miR-151a-5p, miR-17-3p*, and *miR-125b* (*miR-125b-5p*) (Fig. 4e). Among these 9 candidate miRNAs, only *miR-100* and *miR-452* mimics were found to suppress *LINC00589*-driven luciferase activity (Fig. 4f). Therefore, we pursued these two miRNAs as candidates for further investigation.

To verify that *miR-100* and *miR-452* can interact with *LINC00589*, we designed reporter constructs in which the putative *miR-100* and *miR-452*-binding sites in *LINC00589* were mutated by site-directed mutagenesis (Fig. 4g). As expected, *miR-100* and *miR-452* decreased the luciferase activity encoded by the WT *LINC00589* vector, whereas, mutations of the binding sites abolished their suppressive effect (Fig. 4h, i). To verify that these miRNAs directly bind *LINC00589*, we performed MS2 pull-down assays using lysates from WT cells and qRT-PCR confirmation (Supplementary Fig. 8a, b). MS2-*LINC00589* precipitated *miR-100* and *miR-452*, but MS2-*LINC00589*-Mut (with mutation of *miR-100* and *miR-452* binding sequences) failed to enrich these miRNAs, thus suggesting that *LINC00589* directly binds to *miR-100* and *miR-452* through complementary sequences (Fig. 4j). In addition, *LINC00589* overexpression decreased levels of *miR-100* and *miR-452* in TR cells but *LINC00589* knockdown increased levels of *miR-100* and *miR-452* in WT cells (Fig. 4k, l). Altogether, these data indicate that *LINC00589* serves as a platform to sponge *miR-100* and *miR-452* in breast cancer cells.

### *MiR-100* and *miR-452* concurrently regulate trastuzumab resistance, cancer stem cell-like properties, and multidrug resistance

**Fig. 5:**
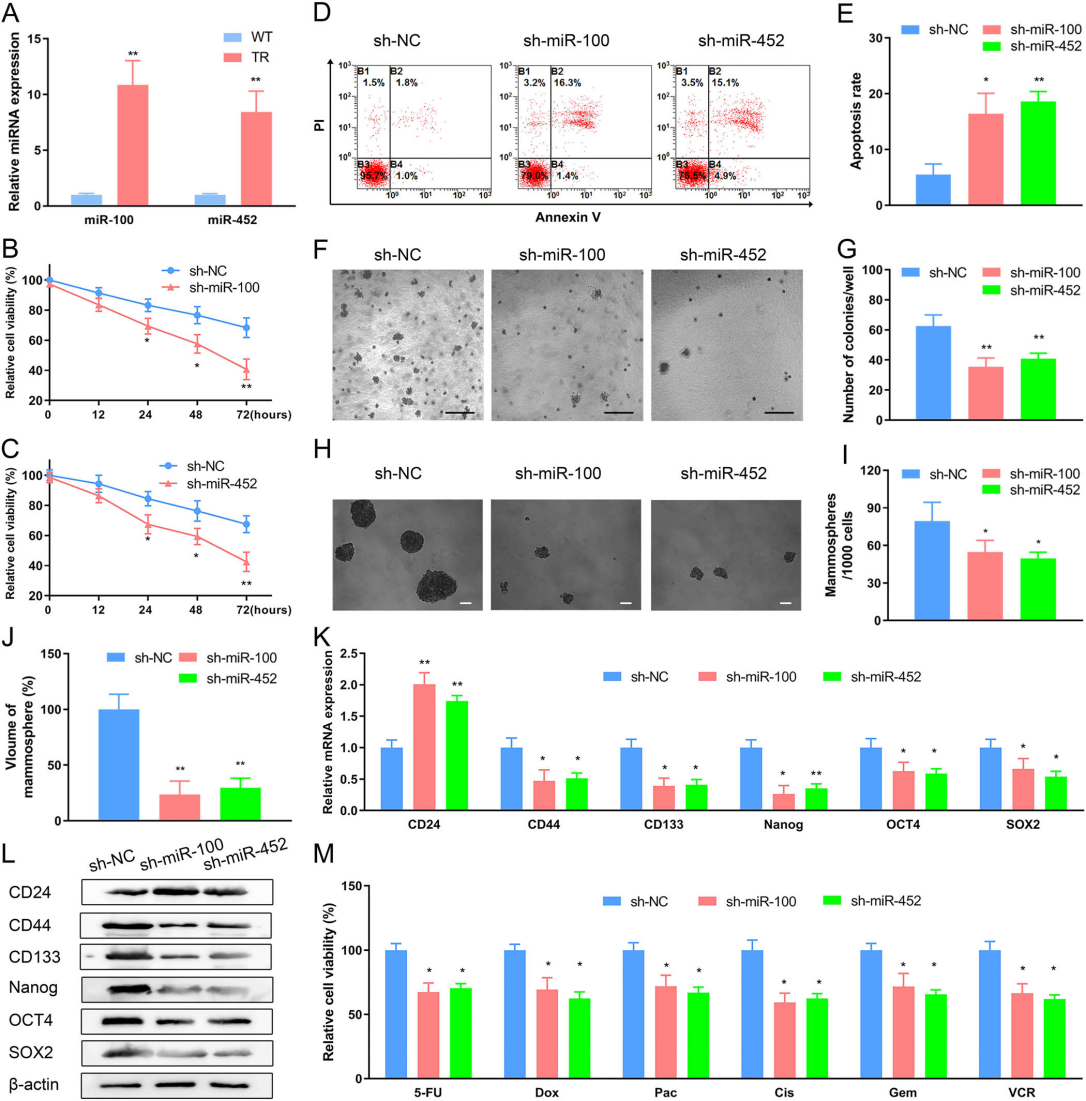
Knockdown of *miR-100* and *miR-452* diminishes trastuzumab resistance, CSC-like properties, and multiple chemoresistance. **A** Expression of *miR-100* and *miR-452* in WT and TR cells were quantified by qRT-PCR assay and then compared. **B**–**E** TR cells were transfected with sh-*miR-100* and sh-*miR-452* and cultured under trastuzumab treatment for 48 h. The cell viability was evaluated using CCK-8 assay (**B**, **C**). The apoptosis was estimated by flow cytometry assay (**D**) and the apoptosis rate was calculated (**E**). **F**, **G** TR cells infected with sh-NC, sh-*miR-100,* and sh-*miR-452* were subjected to soft agar colony formation assay for 21 days. Represent images (**F**) and quantification of the clones (**G**) are shown. Scale bar 100 μm. **H**–**J** *MiR-100*- and *miR-452*-deficient TR cells exhibited decreased mammosphere-formation ability in ultra-low-attachment culture environments. Represent images (**H**), quantification of clones (**I**) and volumes (**J**) are displayed. Scale bar 100 μm. Data are shown as the mean ± SD of five random high-power fields (HPF). **K**, **L** Expression of molecular CSC markers in breast cancer were determined by qRT-PCR (**K**) and western blotting assay (**L**), respectively. **M** *MiR-100*-, *miR-452*-, and the NC-deficient TR cells were exposed to 5-FU, Dox, Pac, Cis, Gem, or VCR for 48 h, followed by CCK-8 assay. Data are shown as mean ± SD; Two-way ANOVA were used to analyze the data in (**B**, **C**) and two-tailed *t* test was used to analyze the data in (**A**, **G**, **I**, **J**, **K**, and **M**). *P < 0.05 and **P < 0.01 versus negative control (NC).

Next, we explored the functional roles of *miR-100* and *miR-452*, which are directly sponged by *LINC00589* in HER2-positive breast cancer cells. qRT-PCR data suggest that expression of both *miR-100* and *miR-452* is much higher in TR cells than in WT cells (Fig. 5A). To determine whether *miR-100* and *miR-452* may also modulate trastuzumab resistance, we constructed lentiviruses expressing shRNAs for *miR-100* and *miR-452* (Supplementary Fig. 9a, b). After trastuzumab treatment, *miR-100* or *miR-452* knockdown suppressed the viability of TR cells (Fig. 5B, C) and increased the apoptosis rate of TR cells (Fig. 5D, E). In addition, soft agar colony formation assays revealed that the anchorage-independent growth of TR cells with *miR-100* or *miR-452* knockdown were dramatically lower than those control groups (Fig. 5F, G). These results suggest that *miR-100* and *miR-452* are involved in trastuzumab resistance in breast cancer.

Further, we investigated the functional roles of *miR-100* and *miR-452* in CSC-like properties and multiple chemoresistance of HER2-positive breast cancer. The mammosphere-formation data showed that the numbers and volumes of spheres were decreased in TR cells infected with sh-*miR-100* and sh-*miR-452* lentiviruses as compared to sh-NC lentivirus (Fig. 5H–J). Consistently, sh-*miR-100* and sh-*miR-452* upregulated the negative stemness marker CD24, and downregulated the positive stemness markers CD44, CD133, Nanog, OCT4, and SOX2, at both the mRNA and protein expression levels (Fig. 5K, L). Finally, the sensitivities of TR cells to 5-Fu, Dox, Paclitaxel, Cisplatin, Gemcitabine and VCR were each increased by *miR-100* and *miR-452* knockdown (Fig. 5M). Taken together, the above results suggest that *miR-100* and *miR-452* concurrently regulate trastuzumab resistance, CSC-like properties and multiple chemoresistance in HER2-positive breast cancer cells.

### *DLG5* and *PRDM16* are direct targets of *miR-100* and *miR-452* and are indirectly regulated by *LINC00589*

**Fig. 6:**
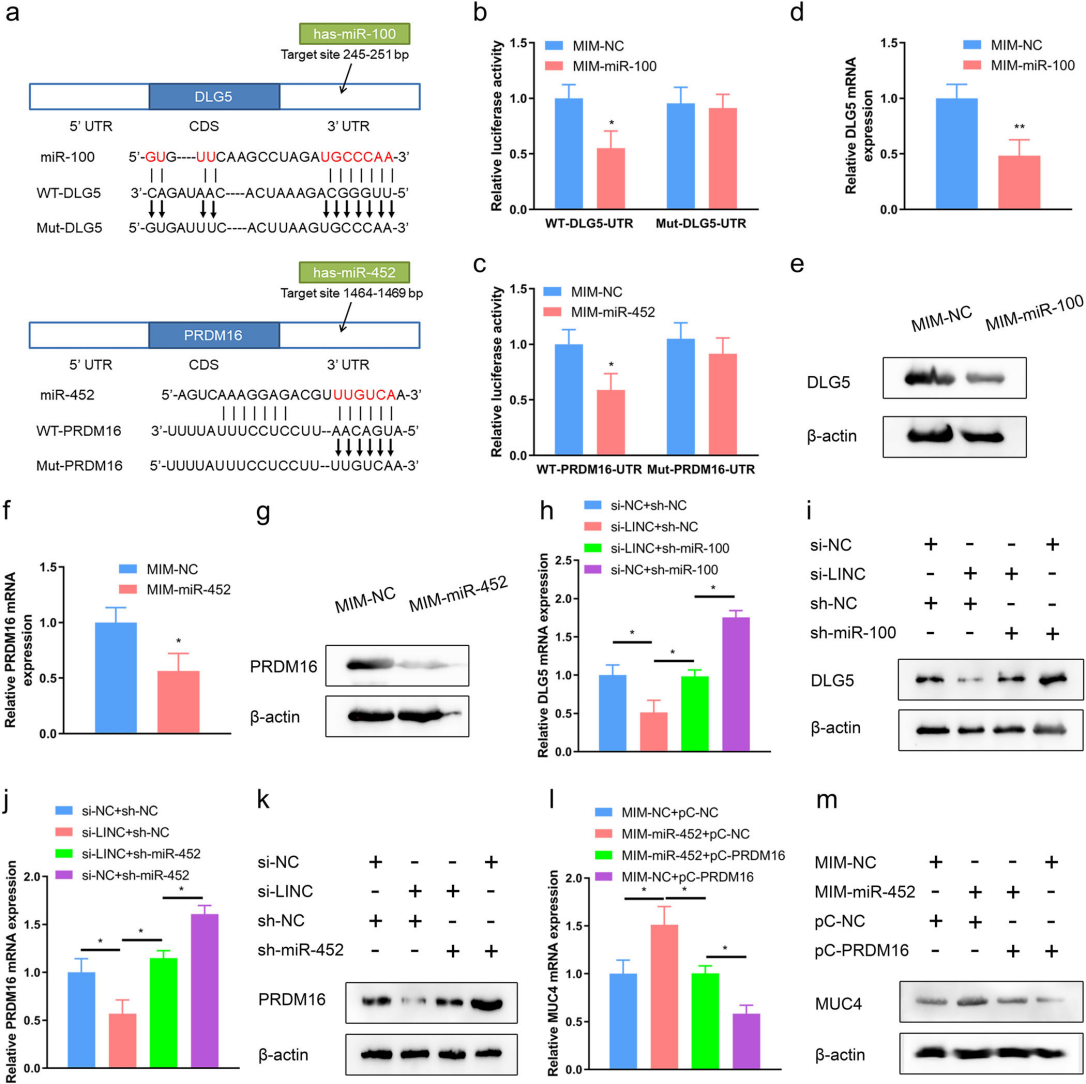
*MiR-100* and *miR-452* directly target *DLG5* and *PRDM16*, which are indirectly regulated by *LINC00589*. **a** Predicted *miR-100* and *miR-452* binding sites within the 3′-UTRs of *DLG5* (upper panel) and *PRDM16* (lower panel), and their mutants containing mutated nucleotides. **b**, **c** WT cells were co-transfected with wild-type (WT) or mutant (Mut) reporter plasmids; and NC mimic (MIM), *miR-100* (**b**), or *miR-452* (**c**) for 48 h, and then subjected to luciferase reporter assay. Renilla luciferase was used as an internal control. **d**–**g** WT cells were transfected with NC, *miR-100* or *miR-452* MIM for 48 h. qRT-PCR assay and western blotting assay were used to test mRNA and protein expression of *DLG5* (**d**, **e**) and *PRDM16* (**f**, **g**). GAPDH and β-actin were used as the internal control, respectively. **h**–**m** WT cells were subjected to co-transfection with siRNAs, shRNAs or pCDNA vectors as indicated for 48 h. mRNA and protein expression of *DLG5* (**h**, **i**), *PRDM16* (**j**, **k**) and *MUC4* (**l**, **m**) were measured using qRT-PCR and western blotting assays. Data are shown as mean ± SD; two-tailed *t* test was used to analyze the data in (**b**, **c**, **d**, **f**, **h**, **j**, and **l**). *P < 0.05 and **P < 0.01 versus negative control (NC).

The biological functions of miRNAs rely on their targets that could be bound with and silenced via the RNA-induced silencing complex. Haven demonstrated the ceRNA interactions of *LINC00589*-*miR-100* and *LINC00589*-*miR-452*, We sought to identify downstream targets of *miR-100* and *miR-452* to elucidate the mechanism through which *LINC00589* exerted its functions in TR cells. First, we identified potential targets from the human genome using the bioinformatic miRNA target prediction tools, including miRwalk, Starbase, RNA22, PITA, miRDB, and TargetScan. Among the predicted targets, Discs large homolog 5 (*DLG5*) for *miR-100* and PR/SET domain 16 (*PRDM16*) for *miR-452* drawed our attention for the following reasons. *DLG5* has been reported to be a suppressor of CSC-like properties in breast cancer cells^22^; and *PRDM16* functions as a tumor suppressor by inhibiting the transcription of *mucin4* (*MUC4*), which is associated with trastuzumab resistance and CSC-like properties of cancer cells^23,24^. To validate these predicted targets for *miR-100* and *miR-452*, we constructed wild-type (WT) and mutant (Mut) dual-luciferase reporter vectors for the *DLG5* and *PRDM16* 3′-UTRs (Fig. 6a). qRT-PCR results confirmed that *miR-100* and *miR-452* mimic significantly upregulated the endogenous levels of *miR-100* and *miR-452* in SKBR3 breast cancer cells (Supplementary Fig. 9c, d). Furthermore, dual-luciferase reporter assays showed that *miR-100* and *miR-452* significantly inhibited WT but not Mut *DLG5* and *PRDM16* luciferase activity, respectively (Fig. 6b, c). To further investigate the regulatory effects of *miR-100* and *miR-452* on *DLG5* and *PRDM16* expression, we performed qRT-PCR and western blotting analyses, which revealed that *miR-100* mimic decreases *DLG5* mRNA and protein expression (Fig. 6d, e), and *miR-452* mimic decreases *PRDM16* mRNA and protein expression (Fig. 6f, g). These results suggest that *DLG5* and *PRDM16* are direct targets for *miR-100* and *miR-452*.

Next, we investigated whether *LINC00589* regulates *DLG5* and *PRDM16* via *miR-100* and *miR-452*. Knockdown of *LINC00589* decreased *DLG5* expression, whereas *miR-100*-suppression abolished the inhibitory effect of *LINC00589* silencing on *DLG5* expression in WT cells, as confirmed at both the mRNA (Fig. 6h) and protein levels (Fig. 6i). Furthermore, knockdown of *LINC00589* suppressed *PRDM16* expression, which was rescued by *miR-452* inhibition (Fig. 6j, k). These results suggested that *LINC00589* regulated *DLG5* and *PRDM16* via *miR-100* and *miR-452,* respectively. Further, we constructed *PRDM16* overexpression vector to confirm the regulatory effect of *PRDM16* on *MUC4* levels in breast cancer cells, and the efficiency was tested by qRT-PCR assay (Supplementary Fig. 10a). *MiR-452* mimic enhanced *MUC4* expression, whereas *PRDM16* overexpression abolished the *miR-452*-induced upregulation of *MUC4* in WT cells (Fig. 6l, m). These results provide further evidence for *MUC4* as a downstream target in the *LINC00589*-*miR-452*-PRMD16 axis. Collectively, our results identified the *LINC00589*-*miR-100*-*DLG5* and the *LINC00589*-*miR-452*-*PRDM16*-*MUC4* axes in breast cancer.

### *LINC00589* regulates trastuzumab resistance, cancer stem cell-like properties, and multiple chemoresistance of breast cancer via *DLG5* and *PRDM16*

**Fig. 7:**
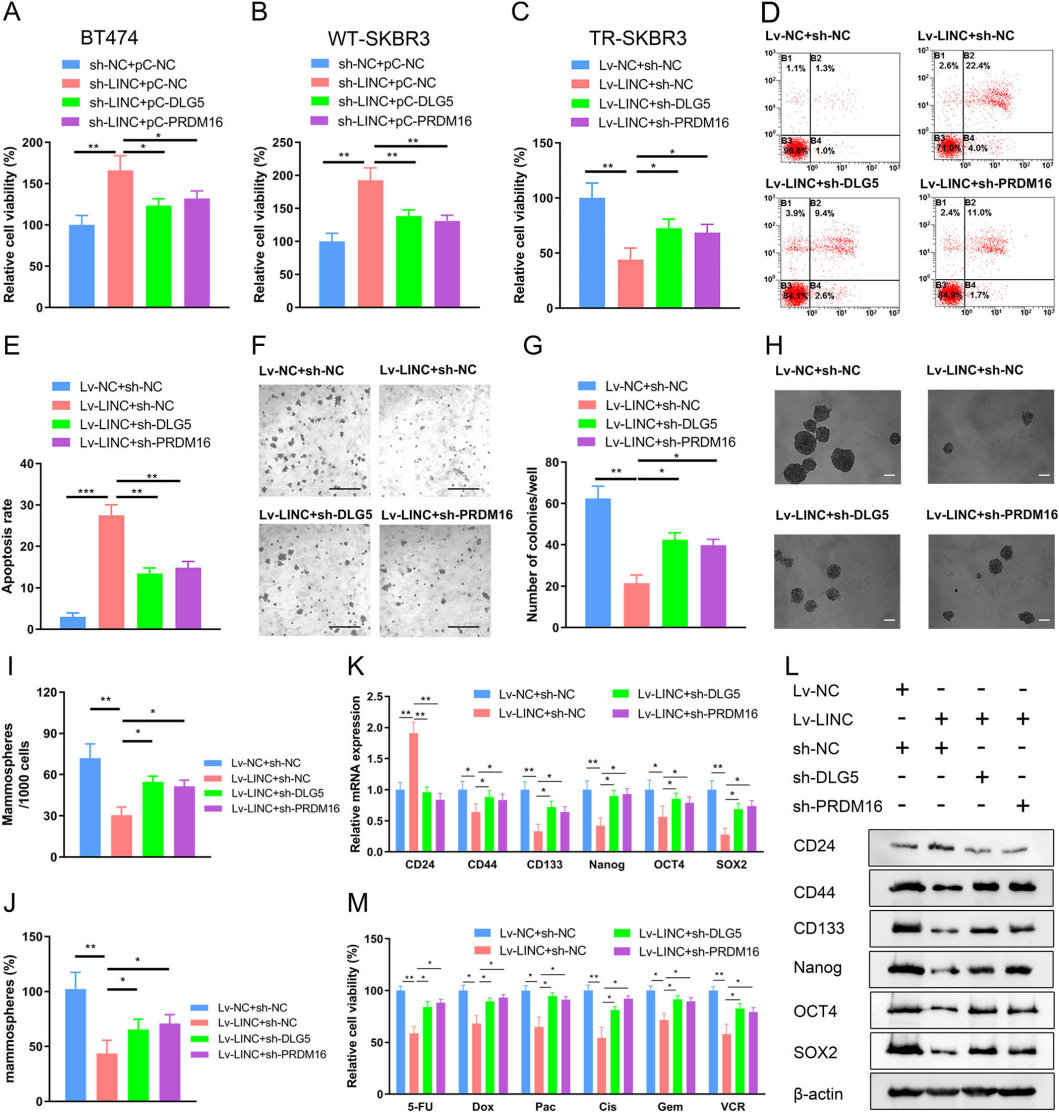
*LINC00589* represses trastuzumab resistance, cancer stem cell-like properties, and multiple chemoresistance via *DLG5* and *PRDM16*. **A**–**E** WT SKBR3 and BT474 cells infected with sh-*LINC00589* or sh-NC lentivirus were transfected with pCDNA (pC) -*DLG5* or pC-*PRDM16* for 48 h; or TR SKBR3 cells infected with Lv-*LINC00589* or the Lv-NC were transfected with sh-*DLG5* or sh-*PRDM16* for 48 h. The relative cell viabilities of WT SKBR3 cells (**A**), BT474 cells (**B**) and TR SKBR3 cells (**C**) were quantified by CCK-8 assay. Apoptosis of TR cells were examined by flow cytometry assay (**D**) and the apoptosis rate was calculated (**E**). **F**–**M** *LINC00589*- or NC- overexpressing TR cells were infected with sh-NC, sh-*DLG5* or sh-*PRDM16* lentivirus. Cells were cultured in soft agar for 21 days, representative images of colony formation were observed (**F**), and the number of colonies were calculated (**G**). Scale bar 100 μm. Cells were seeded in an ultra-low-attachment culture system. Represent images of mammosphere formation were observed (**H**), and numbers (**I**) and volumes (**J**) of mammospheres were calculated. Scale bar 100 μm. Data are shown as the mean ± SD of five random high-power fields (HPF) and were analyzed by two-tailed *t* test. *P < 0.05, and **P < 0.01. mRNA and protein expression of molecular markers of breast cancer CSCs were determined by qRT-PCR assay (**K**) and western blotting (**L**). GAPDH and β-actin were used as internal controls. **M** Cells were exposed to 5-FU, Dox, Pac, Cis, Gem, or VCR for 48 h, and the cell viability was evaluated by CCK-8 assay. Data are shown as mean ± SD; two-tailed *t* test was used to analyze the data in (**A**, **B**, **C**, **E**, **K**, and **M**). *P < 0.05, **P < 0.01, and ***P < 0.001 versus negative control (NC).

We next investigated whether *LINC00589* exertes its biological functions through *DLG5* and *PRDM16*. Overexpression efficacy of pCDNA-*DLG5* (Supplementary Fig. 10b), and knockdown efficacy of sh-*PRDM16* and sh-*DLG5* were determined by qRT-PCR (Supplementary Fig. 10c, d). The CCK-8 assays demonstrated that knockdown of *LINC00589* increased the viability of WT SKBR3 and BT474 cells under trastuzumab treatment, but that overexpression of either *DLG5* or *PRDM16* abolished the increased cell viability induced by sh-*LINC00589* (Fig. 7A, B). In contrast, the viability of TR cells was suppressed by *LINC00589*, while sh-*DLG5* or sh-*PRDM16* co-transfection abolished the decreased cell viability mediated by *LINC00589* (Fig. 7C). Consistently, knockdown of *DLG5* and *PRDM16* also partially abolished *LINC00589*-enhanced cell apoptosis after trastuzumab treatment (Fig. 7D, E). Moreover, soft agar colony formation assays revealed that silencing of *DLG5* or *PRDM16* abrogated *LINC00589*-induced inhibition of anchorage-independent growth in TR cells (Fig. 7F, G). These data indicate that *LINC00589* reverses trastuzumab resistance by regulating *DLG5* and *PRDM16*.

To confirm the roles of *DLG5* and *PRDM16* in *LINC00589*-regulated CSC-like properties and multiple chemoresistance of breast cancer, we performed mammosphere formation and cell viability assays. The number and volume of spheres were downregulated by *LINC00589* in TR cells, while knockdown of either *DLG5* or *PRDM16* partially abated the suppressive effect of *LINC00589* (Fig. 7H–j). Furthermore, qRT-PCR and western blot analyses revealed that knockdown of *DLG5* or *PRDM16* partially relieved the stimulatory effect of *LINC00589* on the expression of the negative stemness marker, CD24, and antagonized the repression of *LINC00589* on the expression of the positive stemness markers, CD44, CD133, Nanog, OCT4, and SOX2 (Fig. 7K, L). Finally, cell viability assays demonstrated that silencing of *DLG5* or *PRDM16* partially abrogated *LINC00589*-induced sensitization of TR cells to 5-FU, Dox, Pac, Cis, Gem, and VCR. (Fig. 7M). Collectively, these results indicate that *LINC00589* regulates trastuzumab resistance, cancer stem cell-like properties and multiple chemoresistance, at least in part, by modulating *DLG5* and *PRDM16* expression.

### *LINC00589*-initiated ceRNA networks serve as key regulators of trastuzumab resistance in vivo

**Fig. 8:**
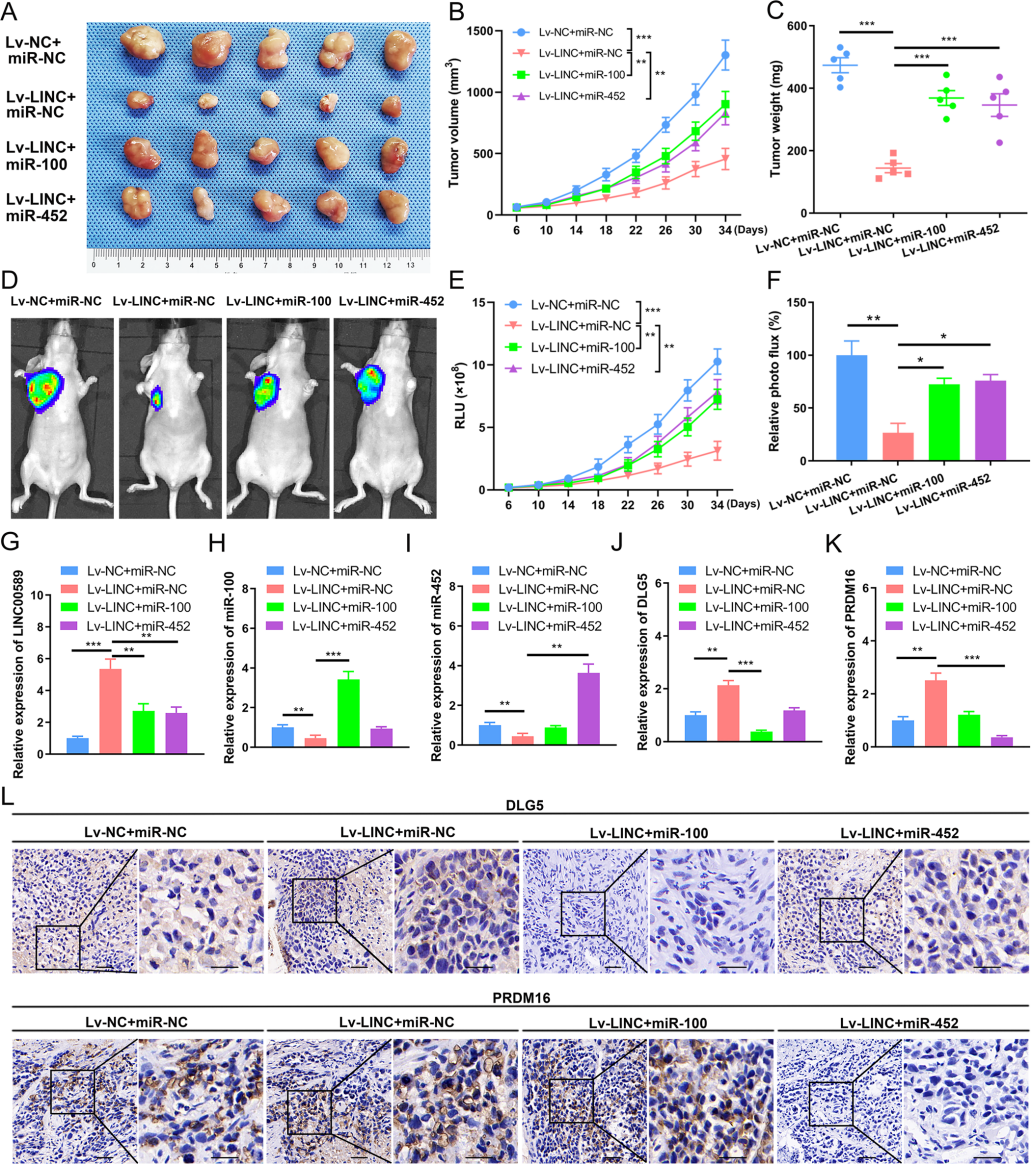
Function of *LINC00589* initiated ceRNA networks in vivo. **A** Nude mice were subcutaneously injected with different TR breast cancer cells with different treatments, including Lv-NC, Lv-*LINC00589*, miR-NC, *miR-100* mimic, or *miR-452* mimic, (*n* = 5). Images of tumors dissected from four groups of nude mice at the end of the experiment are shown. **B** The tumor volume was recorded at the indicated days. **C** Final weights of tumors resected from all groups of sacrificed mice. **D**–**F** Nude mice were implanted with the indicated TR cells (*n* = 5). Whole-body fluorescent images were obtained at the indicated time intervals after injection (**D**), the luciferase activity at different times (**E**) and at the end (**F**) was calculated. Data are represented as mean ± SD and were analyzed by two-way ANOVA. **G**–**K** Expression of *LINC00589* (**G**), *miR-100* (**H**), *miR-452* (**I**), *DLG5* (**J**), and *PRDM16* (**K**) in tumor tissues from the xenografts were determined by qRT-PCR assay. **L** Protein expression of DLG5 and PRDM16 in tumor tissues from the xenografts were analyzed by immunohistochemistry (IHC). Represent images are shown. Scale bar 50 μm. Data are shown as mean ± SD; two-tailed *t* test was used to analyze the data in (**J**, **K**). *P < 0.05, **P < 0.01, and ***P < 0.001 versus negative control (NC).

To investigate the functional role of the *LINC00589*-initiated ceRNA networks in trastuzumab resistance in vivo, we established an animal model of nude mice bearing TR breast cancer cell xenografts. TR cells infected with Lv-*LINC00589* or Lv-NC were implanted into mammary fat pads of nude mice, which were divided into four groups: group A (Lv-NC + miR-NC), group B (Lv-*LINC00589* + miR-NC), group C (Lv-*LINC00589* + *miR-100*) and group D (Lv-*LINC00589* + *miR-452*). When the xenograft volumes reached 50 mm^3^, *miR-100* mimic, *miR-452* mimic or control miRNA were injected into the tumors (15 μg /injection, twice a week) before injection of trastuzumab (10 mg/kg). Consistent with our in vitro results, *LINC00589* overexpression significantly decreased the tumor volume and weight; however, either *miR-100* or *miR-452* reversed the repressive effect of *LINC00589* on tumor volume and weight in nude mice (Fig. 8A–C). To further validate the effect of *LINC00589* on trastuzumab resistance in vivo, we injected TR cells after stable transfection of lentivirus-NC-Luc or lentivirus-*LINC00589*-Luc. The results demonstrate that *LINC00589* inhibited the luciferase activity of tumors, but both *miR-100* and *miR-452* abated *LINC00589*-induced suppression of tumor growth (Fig. 8D–F). These in vivo data suggested that *LINC00589* reversed trastuzumab resistance via *miR-100* and *miR-452* in breast cancer in vivo.

Next, we explored the downstream activation of *LINC00589*-initiated ceRNA networks in vivo. Cancer tissues from xenografts were dissected and subjected to RNA isolation and qRT-PCR. Compared to the control vector, *LINC00589* decreased the mRNA expression of *miR-100*, *miR-452*, and increased the mRNA expression of *LINC00589*, *DLG5* and *PRDM16*. However, *miR-100* abolished the effect of *LINC00589* in promoting mRNA expression of *DLG5*, and *miR-452* abated the enhancement of *LINC00589* on mRNA expression of *PRDM16* (Fig. 8J, K). Moreover, IHC assay data in xenograft tumor tissues revealed that *LINC00589* overexpression could upregulate the protein expressions of DLG5 and PRDM16, while *miR-100* and *miR-452* abrogated the promotion of *LINC00589* on the DLG5 and PRDM16, respectively (Fig. 8L). Collectively, these results confirm the *LINC00589*-initiated ceRNA networks and their downstream targets in vivo.

### *LINC00589*-initiated ceRNA networks are clinically relevant in HER2-positive breast cancer in the clinic

**Fig. 9:**
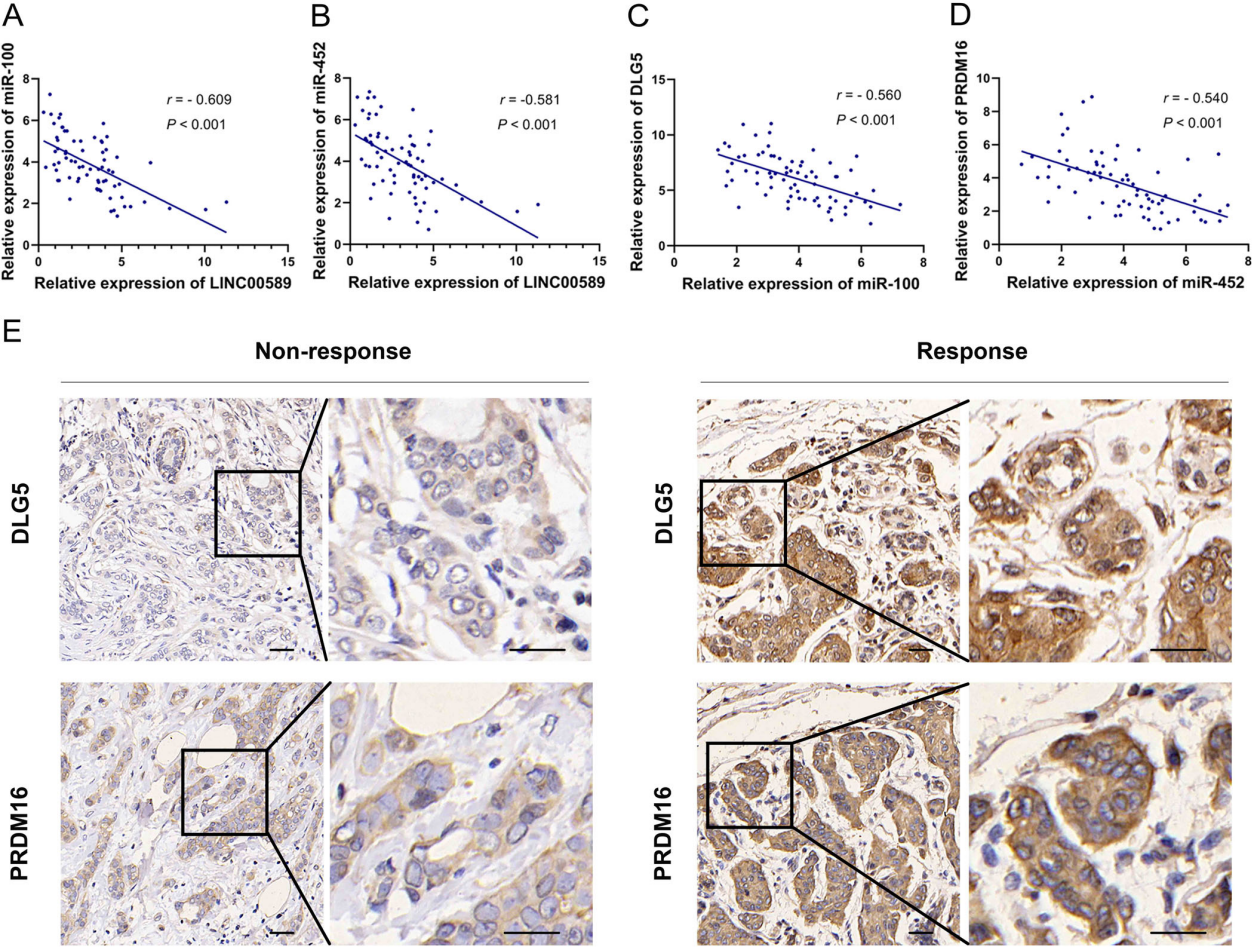
*LINC00589*-dominated regulatory networks in breast cancer patients’ tissues. **A**–**D** mRNA expression of *LINC00589*, *miR-100*, *miR-452*, *DLG5*, and *PRDM16* of the dissected tissues from 71 patients were measured by qRT-PCR assay. The correlation of *LINC00589*-*miR-100* (**A**), *LINC00589*-*miR-452* (**B**), *miR-100*-*DLG5* (**C**), and *miR-452*-*PRDM16* (**D**) were analyzed by the Spearman correlation test. **E** Protein expression of DLG5 and PRDM16 were analyzed by immunohistochemistry (IHC) in tissues from trastuzumab-responding or non-responding patients. Scale bar 50 μm.

To further evaluate the relevance of the *LINC00589*-*miR-100*-*DLG5* and *LINC00589*-*miR-452*-*PRDM16*-*MUC4* networks in clinical samples, we evaluated expression levels in HER2-positive breast cancer patients. The mRNA levels of *LINC00589* were negatively correlated with mRNA levels of both *miR-100* and *miR-452*, and mRNA levels of *miR-100* and *miR-452* were conversely correlated with *DLG5* and *PRDM16*, respectively (Fig. 9A–D). Moreover, IHC assay demonstrated low expression of DLG5 and PRDM16 in trastuzumab non-response HER2-positive breast cancer patients but high expression of DLG5 and PRDM16 in trastuzumab-response HER2-positive breast cancer patients (Fig. 9E). These results confirmed the clinical relevance of the *LINC00589*-initiated ceRNA networks in breast cancer patients in clinic.

## Discussion

**Fig. 10:**
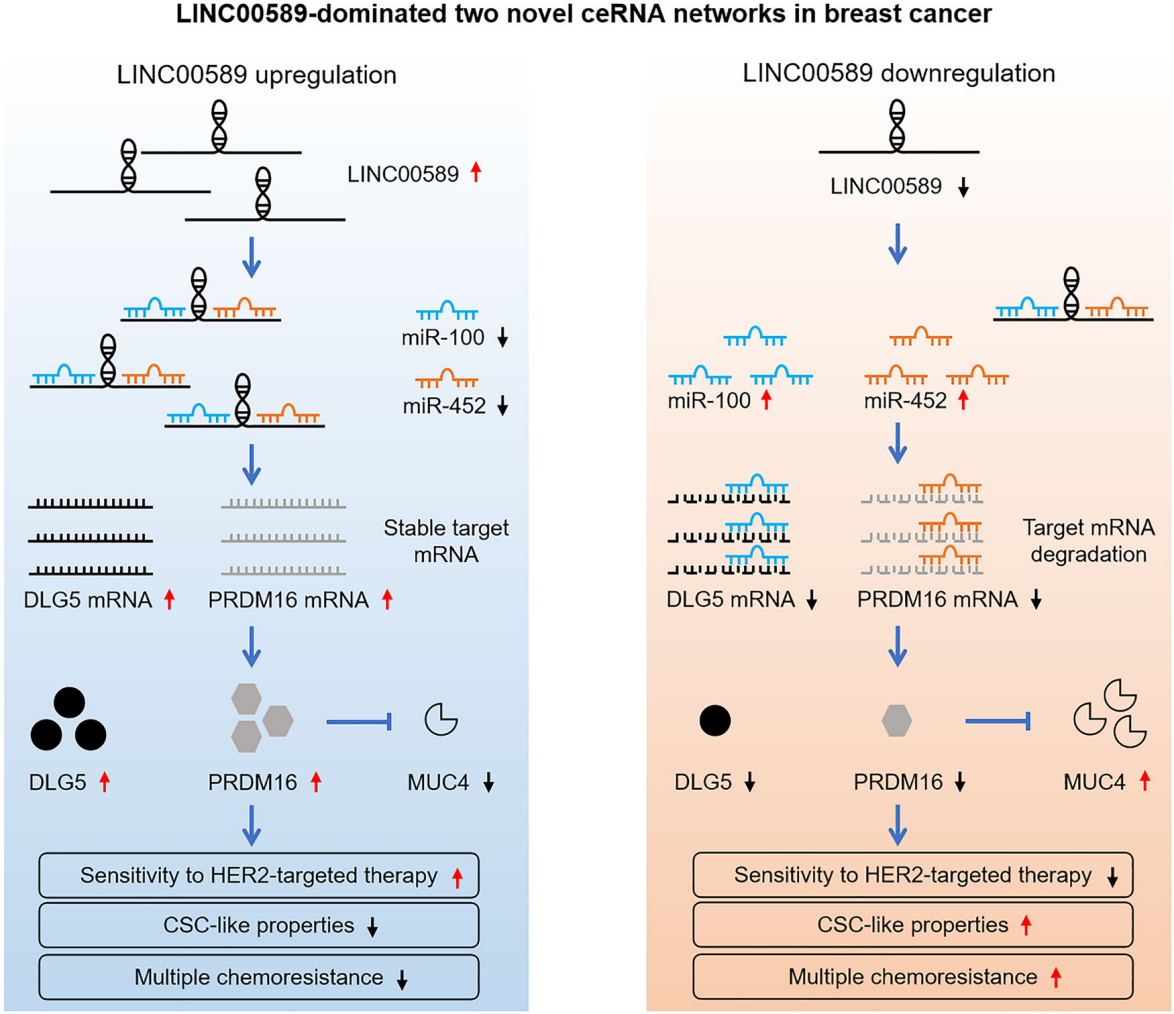
Two *LINC00589*-centered ceRNA networks in breast cancer. LncRNA *LINC00589* serves as a ceRNA platform by sponging *miR-100* and *miR-452* and thereby relieves their repression of *DLG5* and *PRDM16*, which inhibits *MUC4* transcription, resulting in counteractions of trastuzumab resistance, CSC-like properties, and multiple chemoresistance in HER2-positive breast cancer.

Increasing evidence has suggested that drug resistance, EMT and CSC-like properties, which are important causes of cancer progression, may function concordantly. Therefore, the identification of molecular signatures that concurrently regulate these processes holds great significance for cancer characterization, therapy and prognosis evaluation. Here, we provided the evidence that *LINC00589* concurrently reverses trastuzumab resistance, MDR and CSC-like properties and serves as an independent prognostic factor in HER2-positive breast cancer. Further mechanistic investigation revealed that *LINC00589* exerts its functions via two axes, the *miR-100*/*DLG5* and *miR-452*/*PRDM16* axis, as ceRNA platforms (Fig. 10). These data uncover new signaling networks that underlie the crosstalk between trastuzumab resistance, MDR and CSC-like properties in breast cancer.

HER2-targeted therapy is a standard treatment for early or metastatic HER2-positive breast cancer and often improves clinical outcomes; however, primary and acquired resistance occurs in a substantial subset of patients. Many efforts have been made to elucidate the mechanisms of trastuzumab resistance, mainly including but not limited to: (a) HER2 loss, extracellular domain-deficient P95 HER2 expression and MUC4 masking have been demonstrated to block the access of trastuzumab to HER2^25^. (b) High expression of the Delta16 HER2 isoform was shown to mediate optimal efficacy for trastuzumab^26^. (c) Activation of downstream effectors of HER2 signaling (e.g., PTEN) and alternative signaling pathways (e.g., IGF1R signaling) lead to trastuzumab resistance in breast cancer^27^. (d) Breast cancer cells have been demonstrated to escape from antibody-dependent cell-mediated cytotoxicity (ADCC) caused by trastuzumab^28^. These findings imply that trastuzumab resistance may arise from a combination of largely unknown mechanisms. In addition, emerging evidence shows that trastuzumab resistance is not an isolated phenomenon but is often accompanied by MDR and CSC-like properties^18^. However, other potential key regulators that link these processes and their relationships have not been well explored.

Noncoding RNAs, most of which have not been functionally annotated, play central roles in various physiological and pathological processes, especially in complicated signaling networks of cancers^29,30^. Our previous studies and other reports demonstrate that noncoding RNAs also regulate trastuzumab resistance to breast cancer, e.g., miR-200c, miR-221, miR-375, lncRNA TINCR, AGAP2-AS1, and UCA1^1,3,10,11,29,30^. *LINC00589*, also named as TSLNC8, was identified on chromosome 8p12 by Strausberg et al.^31^. Until recently, functional roles of *LINC00589* began to get much concern. For example, *LINC00589* serves as a tumor suppressor in human glioma^32^ and non-small cell lung cancer^15^, and inhibits melanoma resistance to BRAF inhibitor^33^. However, *LINC00589* also interacts with HUR and stabilizes CTNNB1 mRNA, thereby promoting cancer progression in pancreatic cancer^13^. Thus, *LINC00589* may serve as both a tumor suppressor and a tumor promoter according to different cancer pathological settings. In this study, we determined that *LINC00589* is downregulated in trastuzumab-resistant breast cancer and serves as an independent prognostic factor for HER2-positive patients. Furthermore, *LINC00589* concurrently reversed trastuzumab resistance, multiple chemoresistance, and CSC-like properties of HER2 breast cancer. Giusti et al. disclosed that HER2 loss also results in both trastuzumab resistance and enhanced stemness of breast cancer^34^, which is consistent with our findings and indicates HER2 loss probably is likely to correlated *LINC00589*. However, *LINC00589* is unlikely to regulate HER2 expression in breast cancer and instead exerts multiple functions in trastuzumab resistance, MDR and CSC-like properties through a HER2-independent mechanism.

LncRNAs, miRNAs, and mRNAs have been shown to crosstalk with each other through shared binding sequences within complex signaling networks via ceRNA mechanisms^21^. Emerging evidence indicates that lncRNAs serve as sponges for miRNAs and thereby keep them away from binding sites on target genes in various cancer types^21,35^. For example, LncRNA DNACR enhances ROCK1-mediated proliferation and metastasis by sponging miR-335-5p and miR-1972 in osteosarcoma^35^. LncRNA LINC01123 sponges miR-199a-5p and triggers proliferation and aerobic glycolysis by regulating c-myc expression in non-small cell lung cancer^36^. LINC00673, which is upregulated by YY1, exerts oncogenic functions in breast cancer by sponging miR-515-5p and subsequently upregulates MARK4 expression, and inhibits the Hippo signaling pathway^37^. In this study, we demonstrate that *LINC00589* is mainly localized to the cytoplasm, which is consistent with the possibility that it may function as an endogenous miRNA sponge. Bioinformatics analysis and experimental assays further revealed that *miR-100* and *miR-452* are direct targets of *LINC00589*. *MiR-100* and *miR-452* have been reported as both oncogenes and tumor suppressor genes. For example, *miR-100* promotes cetuximab resistance in colorectal cancer^38^ but inhibits bladder urothelial carcinogenesis^39^; and *miR-452* promotes renal cancer cell invasion and metastasis and colorectal cancer progression^40,41^ but inhibits metastasis of non-small cell lung cancer^42^. In our study, downregulation of both *miR-100* and *miR-452* suppressed trastuzumab resistance, multiple chemoresistance, and CSC-like properties, thus supporting their oncogenic roles in HER2-positive breast cancer as targets of *LINC00589*.

According to the ceRNA network theory, roles for lncRNAs are dependent on their abilities to regulate miRNA targets that mediate signaling pathways^37,38^. Therefore, investigating potential targets of miRNAs is important for elucidating the roles and mechanisms of ceRNA networks. Consistently, in this study we identified *DLG5* as a target of *miR-100* and *PRDM16* as a target of *miR-452*. *DLG5* belongs to the membrane-associated guanylate kinase (MAGUK) superfamily and is considered to play multiple roles in various cancers, including an ability to suppress breast cancer stem cell-like characteristics and restore tamoxifen sensitivity by inhibiting TAZ expression^22^ and to decrease the formation and function of invadopodia in human hepatocellular carcinoma via Girdin and Tks5^43^. On the other hand, *PRDM16*, a zinc finger transcription factor hammering the epithelial-to-mesenchymal transition, functions as a suppressor of lung adenocarcinoma metastasis and is associated with patient survival^23^. In kidney cancer, *PRDM16* suppresses HIF-targeted gene expression and inhibits tumor growth in vitro and in vivo^44^. In this study, we identified *DLG5* and *PRDM16* as target genes for *miR-100* and *miR-452*. Thus, our results support the ability of *LINC00589* to regulate both *miR-100*/*DLG5* and *miR-452*/*PRDM16* axes, thereby suggesting two crosslinked ceRNA pathways. In support of this possibility, we demonstrated that silencing of either *DLG5* or *PRDM16* abolished multiple *LINC00589*-induced effects in HER2-positive breast cancer.

In conclusion, we demonstrated that *LINC00589* concurrently reverses trastuzumab resistance, multiple chemoresistance and CSC-like properties and acts as an independent prognosis factor for HER2-positive breast cancer. Further, we identified that two ceRNAs networks, *LINC00589*-*miR-100*-*DLG5* axis and *LINC00589*-*miR-452*-*PRDM16* axis, that mediate multiple suppressor roles of *LINC00589*. Our findings suggest that these *LINC00589* ceRNA networks could be valuable for predicting trastuzumab efficacy and prognosis, as well as providing promising therapeutic targets for HER2-positive breast cancer in future translational applications.

## Methods

### Patient samples

A total of 71 cases of trastuzumab-treated HER2-positive breast cancer patients were enrolled from General Hospital of Xinjiang Command and Xijing Hospital before chemotherapy was initiated. Cases with complete response (CR) or partial response (PR) were considered as trastuzumab responders, and cases with stable disease (SD) or progressive disease (PD) were defined as trastuzumab non-responders. Clinical tissue samples were obtained during the operation and were immediately frozen at −80 °C until RNA extraction. Another independent cohort of 92 cases of paraffin-embedded samples from HER2-positive breast cancer patients who received trastuzumab were obtained from the General Hospital of Xinjiang Command and Xijing Hospital. Ethical approval was obtained from the Ethics Committee of the General Hospital of Xinjiang Command (number: 201803). All participants provided written informed consent. The detailed clinicopathological characteristics of these paraffin-embedded samples are summarized in Table 1. All patients were pathologically confirmed for diagnosis of HER2-positive breast cancer.

### Reagents

Trastuzumab (Herceptin) was purchased from Roche (Basel, Switzerland) and dissolved in phosphate-buffered saline (PBS). 5-Fluorouracil (5-FU), doxorubicin (Dox), paclitaxel (Pac), cisplatin (Cis), gemcitabine (Gem), and vincristine (VCR) were obtained from Sigma-Aldrich (St Louis, MO, USA).

### Cell lines and cell culture

BT474 human breast cancer cells (HER2-overexpression) were obtained from the American Type Culture Collection (catalog number HTB-20, ATCC) and were cultured in RPMI 1640 supplemented with 10% FBS. Wild-type (WT) SKBR3 human breast cancer cells (catalog number HTB-30, ATCC) were cultured in RPMI 1640 medium supplemented with 10% fetal bovine serum (FBS). Trastuzumab-resistant (TR) SKBR3 cells were established by continuous culture of WT SKBR3 cells in the presence of 5 μg/ml trastuzumab for 6 months in a humidified atmosphere of 5% CO_2_ and 95% air at 37 °C according to our previous reports^1,3^.

### Cell transfection

The full-length coding sequences of *DLG5* and *PRDM16* were amplified and cloned into the pCDNA3.1 overexpression vector (catalog number V79520, Invitrogen, Carlsbad, CA, USA). The control and overexpression vectors were transfected using Lipofectamine 2000 (catalog number 11668019, Invitrogen, Carlsbad, CA, USA) at a 1 μg DNA: 2.5 μl lipofectamine ratio according to the manufacturer’s instructions. miRNA mimics were synthesized by Shanghai Gene Pharma Co, Ltd. The target sequences were as follows: *miR-100* mimic: 5’-AACCCGUAGAUCCGAACUUGUG-3’; *miR-452* mimic: 5’- AACUGUUUGCAGAGGAAACUGA-3’; *miR-7* mimic: 5’ UGGAAGACUAGUGAUUUUGUUGUU-3’; *miR-224* mimic: 5’-UCAAGUCACUAGUGGUUCCGUUUAG-3’; *miR-4288* mimic: 5’-UUGUCUGCUGAGUUUCC-3’; *miR-3926* mimic: 5’-UGGCCAAAAAGCAGGCAGAGA-3’; *miR-151a-5p* mimic: 5’-UCGAGGAGCUCACAGUCUAGU-3’; *miR-17-3p* mimic: 5’-ACUGCAGUGAAGGCACUUGUAG-3’; *miR-125b* mimic: 5’-UCCCUGAGACCCUAACUUGUGA-3’. The working concentrations for miRNA mimics were 30 nM for cell transfection. RNA was transfected into cells using Lipofectamine 2000 according to the manufacturer’s instructions.

### Expression vector construction, lentiviral package, and transduction

Full-length coding sequences of *DLG5* and *PRDM16* were amplified and cloned into pcDNA3.1 vector. Full-length coding sequences of *LINC00589* were amplified and cloned into pLVX-Puro vector (catalog number PT4002-5, Clontech, CA, USA) with or without the luciferase gene. RNA oligos containing siRNA sequences of *LINC00589*, *DLG5,* and *PRDM16*, or containing inhibitor sequences of *miR-100* or *miR-452* were synthesized and cloned into the shRNA lentiviral vector pLVX-shRNA2 (catalog number PT4052-5, Clontech, CA, USA) with either the puromycin or bleomycin resistance marker gene. The sequences were as follows: siRNA-*LINC00589*: 5’-GGATGACACCTCCATTCAA-3’; siRNA-*DLG5*: 5’-GCTCAAGAGCAGCACATCT-3’; siRNA-*PRDM16*: 5’-CCCACAACTTGCTGGTCAA-3’. *miR-100* inhibitor: 5’-CACAAGUUCGGAUCUACGGGUA-3’; *miR-452* inhibitor: 5’-UCAGUUUCCUCUGCAAACAGTT-3’. The retrovirus constructs or the empty vector (control vector) were transiently co-transfected with package vectors into 293T cells to produce lentiviruses, which were collected in the viral supernatant 72 h after transfection. Cells infected with the packaged viruses were pre-treated with DEAE dextran (25 μg/ml) for 45 min. 48 h after the infection, cells were screened with puromycin (2 μg/ml) or bleomycin (100 μg/ml), depending on the selection marker of the vector. Cells infected with multiple constructs were selected for infection with each construct.

### Bioinformatic analysis

The sequence of *LINC00589* was downloaded from NCBI (gene ID: 619351), from which a 1413–base pair (bp) sequence was extracted. The secondary structure of *LINC00589* was predicted by AnnoLnc (http://annolnc.cbi.pku.edu.cn/)^45^. The lncRNA subcellular localization was predicted using the online website lncLocator (http://www.csbio.sjtu.edu.cn/bioinf/lncLocator/)^46^. The potential sponged miRNAs for *LINC00589* and their binding sites were predicted by LincBase tools (http://carolina.imis.athena-innovation.gr/diana_tools). Starbase (http://starbase.sysu.edu.cn/), miRWalk (http://mirwalk.umm.uni-heidelberg.de/), RNA22 (https://cm.jefferson.edu/rna22/), PITA (https://genie.weizmann.ac.il/pubs/mir07/), miRDB (http://mirdb.org/), and TargetScan (http://www.targetscan.org/vert_71/), were used to predict the potential target genes of *miR-100* and *miR-452*.

### Real-time RT-PCR analysis

Total cell RNA was extracted using Trizol (Invitrogen). cDNA was synthesized from 1.0 μg of total RNA, using oligo-dT priming and the Retroscript reverse transcription kit (Ambion, cat no. AM1710). Real-time PCR was performed in triplicate using the Real-Time SYBR Green PCR master mix system (SuperArray Bioscience Corporation, cat no. PA-110) on an Opticon DNA Monitor instrument (Biorad). mRNA levels were normalized to GAPDH, which was used as an internal control. The primer sequences were as follows: forward primer 5’-CACCTCCATTCAACCAATAAGC-3’ and reverse primer 5’-ACCCTGTCCCCAATAACCC -3’ for *LINC00589*; forward primer 5’-GGTCTCACTCTCTCTTCTGCATCTCT-3’, reverse primer 5’-GGCATCCATCATCTAGTCAAACCTC-3’ for *CD24*; forward primer 5’-CGACAGCACAGACAGAATCCC-3’, reverse primer 5’- AATCAAAGCCAAGGCCAAGAG-3’ for *CD44*; forward primer 5’- AGTCGGAAACTGGCAGATAGC-3’, reverse primer 5’-GGTAGTGTTGTACTGGGCCAAT-3’ for *CD133*; forward primer 5’-AGGCAAACAACCCACTTCTG-3’, reverse primer 5’-TCTGCTGGAGGCTGAGGTAT-3’ for *Nanog*; forward primer 5’-ATGTGGTCCGAGTGTGGTTC-3’, reverse primer 5’-CAGAGTGGTGACGGAGACAG-3’ for *OCT4*; forward primer 5’-AACCAGCGCATGGACAGTTA-3’, reverse primer 5’-GACTTGACCACCGAACCCAT-3’ for *SOX2*; forward primer 5’-CTGCACATCAACCTCAGTGG-3’, reverse primer 5’-CGGCAGCATACACTCCATT-3’ for *DLG5*; forward primer 5’-AACCAAGCATCAACGCGAAC-3’, reverse primer 5’-AACCCTGGTTCTTAGCCTGC-3’ for *PRDM16*; forward primer 5’-TGGGACGATGCTGACTTCTC-3’, reverse primer 5’-CCCCGTTGTTTGTCATCTTTC-3’ for *MUC4*; forward primer 5’-GTTCTCTGCCGTAGGTGTCC-3’, reverse primer 5’-GAACCAGCCAGATGTTCGGC-3’ for *HER2*; and forward primer 5’- CTCCTCCACCTTTGACGCTG-3’, reverse primer 5’-TCCTCTTGTGCTCTTGCTGG-3’ for *GAPDH*. For miRNAs, the expression levels were normalized to U6 small nuclear RNA (internal control), and the following universal primers from the QIAGEN kit were used: forward primer 5’-AACCCGTAGATCCGAACTTGTG-3’ for *miR-100*; forward primer 5’-AACUGUUUGCAGAGGAAACUGA-3’ for *miR-452*; and forward primer 5’-GTGCTCGCTTCGGCAGCACATAT-3’ for *U6*^47^. All quantitative reverse transcription polymerase chain reaction (qRT-PCR) analysis was performed in an ABI Prism 7500 (Applied Biosystems). The expression change was calculated using the 2^−ΔΔ^CT method.

### Immunoblotting assay

The following antibodies were used for western blotting: rabbit monoclonal anti-CD24 (1:500, catalog number ab179821, Abcam, MA, USA), rabbit monoclonal anti-CD44 (1:500, catalog number ab189524, Abcam), rabbit recombinant multiclonal anti-CD133 (1:800, catalog number ab278053, Abcam), mouse monoclonal anti-Nanog (1:1000, catalog number ab173368, Abcam), rabbit monoclonal anti-OCT4 (1:500, catalog number ab200834, Abcam), mouse monoclonal anti-SOX2 (1:1000, catalog number ab79351, Abcam), rabbit polyclonal anti-DLG5 (1:1000, catalog number ab231283, Abcam), rabbit polyclonal anti-PRDM16 (1:1000, catalog number ab106410, Abcam), mouse monoclonal anti-ErbB-2 (1:500, catalog number sc-33684, Santa Cruz Biotechnology, CA, USA), and mouse monoclonal anti-β-actin (1:1000, catalog number A5441, Sigma-Aldrich, MO, USA). Other primary antibodies and secondary antibodies and experimental procedures for immunoblotting are provided in the supplementary experimental procedures^10^.

### Immunohistochemistry (IHC)

Four-micrometer sections from breast cancer paraffin-embedded tissue samples were used to perform IHC staining by PV-6000 detection kits, and ZLI-9032 DAB substrate kit (Beijing Zhongshan golden bridge Biotechnology Co. Ltd., Beijing, China) The sections were incubated in anti-DLG5 antibody (1:300, catalog number ab231283, Abcam) or anti-PRDM16 antibody (1:400, catalog number ab106410, Abcam) at 4 °C overnight. The results of IHC staining were evaluated by an experienced pathologist, and quantification of the reaction was performed using the histoscore system as previously described^48^.

### Luciferase reporter construction

The 3’-untranslated region (3’-UTR) of *DLG5* and *PRDM16* and full-length *LINC00589* were amplified from human genomic DNA by PCR and cloned into a modified pGL3 luciferase vector (catalog number E1751, Promega, Madison, WI, USA). Wild-type (WT) and mutant (Mut) binding sites of *miR-100* in *LINC00589* and the 3’-UTR of *DLG5*, and binding sites for *miR-452* in *LINC00589* and the 3’-UTR of *PRDM16* were subcloned into the pGL3 basic vector to generate corresponding WT and Mut luciferase reporter vectors. Primers for mutation of binding sites between *miR-100* and *LINC00589* were: forward-1, 5’-TATGTCAGAGATGCTAGCACTGGCATC-3’; and reverse-1, 5’-AGGTGGCATTCTAGTGGACACTCTTG-3’, forward-2, 5’-CAAGAGTGTCCACTAGAATGCCACCT-3’; and reverse-2, 5’-GGTTTGAAATGAGAGTGTCAACCTTC-3’. Primers for mutation of binding sites between *miR-452* and *LINC00589* were: forward-1, 5’-GCTGGAAAGTGAGCCTGGATCTCTCT-3’; and reverse-1, 5’-CTGACAAACGTTTTGGGGTTCTCGC-3’, and forward-2, 5’-GCGAGAACCCCAAAACGTTTGTCAG-3’; and reverse-2, 5’-AACCTGAAACTCAGATGGGCAAGATTA-3’. Primers for mutation of binding sites between *miR-100* and *DLG5* were: forward-1, 5’-GAGAATGCTGTGCTGTGGATGAC-3’; and reverse-1, 5’-TTGGGCACTTAAGTGAAATCAC-3’, forward-2, 5’-GTGATTTCACTTAAGUGCCCAA-3’; and reverse-2, 5’-AGGAGAGGTGCCACCAAGGAGCA-3’. Primers for mutation of binding sites between *miR-452* and *PRDM16* were: forward-1, 5’-GTTCTTGGCGAGACACAGCTTGAG-3’; and reverse-1, 5’-TTGACAAAAGGAGGAAATAAAA-3’, forward-2, 5’-TTTTATTTCCTCCTTTTGTCAA-3’; and reverse-2, 5’-TCTTCCAAACAATACAAGAAATA-3’.

### Dual-luciferase reporter gene assays

Breast cancer cells were co-transfected with 150 ng of firefly luciferase reporter plasmid with inserted WT and Mut sequences from *LINC00589*, *DLG5*, or *PRDM16*, together with pRL-SV40 Renilla luciferase vector (catalog number E2231, Promega) and miRNAs (*miR-100* mimic, *miR-452* mimic or negative control RNA) using Lipofectamine 2000 (Invitrogen). Three independent transfection experiments were performed, each in triplicate. 48 h after transfection, firefly luciferase activity derived from pGL3 plasmids was evaluated and normalized to Renilla luciferase activity using a luciferase assay system (Promega) as reported previously^1^.

### CCK-8 cytotoxicity assay

Cytotoxicity was analyzed using the cell counting kit-8 (CCK-8) method (MYBiotech, China). Briefly, breast cancer cells were transfected with plasmids and/or miRNA mimics/shRNAs or were infected with lentiviruses. Subsequently, the cells were plated in 96-well plates and exposed to trastuzumab, 5-FU, doxorubicin, paclitaxel, cisplatin, gemcitabine, or vincristine over a time course. After drug treatment, 10 μl CCK-8 solution was added to each well, and the cells were incubated at 37 °C for 4 h. The optical density (OD) was measured at 450 nm (Thermo Scientific, USA), and the half-maximal inhibitory concentration (IC50) of the drug was calculated based on the OD value. The assay was performed at least three times. Cell cytotoxicity was calculated according to the following formula: inhibition ratio (%) = (OD (drug − OD (blank))/(OD (drug control) − OD (blank)) × 100%^49^.

### Soft agar colony formation assays

Low melting temperature agarose was mixed with culture medium to obtain 0.6% and 0.35% gel as the “lower” and the “upper” soft agar, respectively. Plates with 6 wells were coated with 1.0 ml lower soft agar. Then 1.0 × 10^3^ cells were resuspended with 2 ml upper soft agar and immediately plated on the lower soft agar. Cells were incubated for 21 days in a 37 °C incubator and were stained with 0.005% Crystal Violet Staining Solution. Colonies were enumerated by microscopy. Experiments were carried out in triplicate and were repeated a minimum of three times^50^.

### Flow cytometry analysis

WT SKBR3 (catalog number HTB-30, ATCC) and TR SKBR3 breast cancer cells were seeded in six-well plates (5 × 10^5^ cells/well) and were transfected with miRNA mimics or plasmids, or they were infected with lentiviruses. The cultures were supplemented with trastuzumab at a final concentration of 5 μg/ ml for WT SKBR3 cells (catalog number HTB-30, ATCC) cells or 25 μg/ml for TR SKBR3 cells. Then, the cells were washed three times with PBS, harvested, stained with annexin V-FITC and propidium iodide (BD Biosciences), and subjected to flow cytometry (BD Biosciences) to detect apoptosis^51^.

### Subcellular fractionation assay

Seeded breast cancer cells (2 × 10^7^ cells) were washed with ice-cold PBS and resuspended in the ice-cold cytoplasmic lysis buffer (0.15% NP-40, 10 mM Tris pH 7.5, 150 mM NaCl) for 5 min on ice. The lysates were transferred into ice-cold sucrose buffer and centrifuged at 13,000 × *g* for 10 min at 4 °C. The supernatant (~700 μL) was collected as the cytoplasmic fraction and the precipitate was collected as the nuclear fraction. The expression of *LINC00589* in different subcellular fractionations was analyzed by qRT-PCR^52^.

### Mammosphere-formation assay

Breast cancer cells were seeded onto ultra-lowattachment six-well plates (3471; Corning, Corning, NY, USA) at a density of 2000 cells per well. The CSCs were cultured for 14 days using the MammoCult Human Medium Kit (Stemcell Technologies, Vancouver, BC, Canada) according to the manufacturer’s instructions. Mammospheres were digested in trypsin/EDTA and centrifuged at 300 × *g* for 10 min. Then, the cells were resuspended and cultured for the next round of sphere formation. Cells from the sixth-generation spheres were used to analyze the efficiency of mammosphere formation. Formed spheres were counted manually, and representative images were obtained by microscopy^53^.

### MS2 RNA pull-down assay

To explore the interactions between *LINC00589* and miRNAs, we performed a MS2 RNA pull-down assay, in which the MBP-MCP fusion protein recognizes MS2 hairpins. Breast cancer cells were transfected with MS2, *LINC00589*-MS2 or *LINC00589*-Mut-MS2 plasmids and harvested 48 h post-transfection. Then, breast cancer cell lysates were incubated with MBP-MCP-coated amylase resin (prepared at 4 °C) for 8 h. Bound *LINC00589*-MS2 complexes were eluted with 100 μl buffer containing 20 mM maltose after incubation and extensive washing. The eluted complexes were used to identify *LINC00589*-associated miRNAs. qRT-PCR analysis was performed to identify the miRNAs associated with *LINC00589*^54^.

### Tumor xenografts and growth measurement

TR breast cancer cells were infected with *LINC00589* overexpression or control lentivirus and cultured for cell expansion. Female athymic BALB/c nude mice (4–6 weeks, 20 g) were purchased from the Experimental Animal Center, Chinese Academy of Science (Shanghai, China). Mice were housed in a pathogen-free animal facility at 22 ± 2 °C under controlled 12-h light/dark cycles. Mice were given regular chow or special custom diets when indicated and had access to autoclaved water ad libitum. Animals were grouped by simple randomization using a random number table. To form orthotopic mammary fat pad tumors, the surgical area was depilated and swabbed with 70% ethanol before making an incision in the skin of the breast. Next, 4 × 10^6^ cells were subcutaneously injected into the mammary fat pad area in situ. When the volume of xenograft tumors reached 50 mm^3^, *miR-100* mimic, *miR-452* mimic or control miRNA mimic complexed with a lipid-based delivery agent (15 μg/injection, twice a week) were injected into the tumors 72 h prior to intravenous injection of trastuzumab (10 mg/kg, twice a week). Tumor volumes were monitored every 3 days for 7 weeks according to the formula: tumor volume (mm^3^) = length × width^2^/2. To further investigate the function of *LINC00589*-initiated ceRNA networks in vivo, another group of nude mice were injected with *LINC00589*-Luc and control Luc lentivirus-infected TR breast cancer cells and were administered the same treatments described above. Five minutes after administration of 1.5 mg luciferin (Gold Biotech, St Louis, MO, USA), the luciferase activity of tumor xenografts was monitored using an IVIS@ Lumina II system (Caliper Life Sciences, Hopkinton, MA, USA), which was repeated every 3 days. At the end of the experiments, all mice were given euthanasia by amobarbital injection of three times standard doses, and the tumor tissues were isolated and snap-frozen for mRNA expression analysis. The investigators had no bias and special tendency in the processing of animal experiments. All animal experiments were performed according to the guidelines of the Institutional Animal Care and Use Committee of the General Hospital of Xinjiang Command and were approved by the local animal experiments ethical committee.

### In situ hybridization (ISH) staining assay

Expression of *LINC00589* in paraffin-embedded breast cancer tissues was determined by in situ hybridization (ISH) experiments. Briefly, after dewaxing and rehydration, the paraffin-embedded breast cancer tissues were digested with 10% trypsin for 40 min at room temperature and then were hybridized with the digoxin-modified *LINC00589* probe (5’-TACTGTCTCTCCTCGGAGCAGGATTCCATCTTT-3’, Exiqon, Vedbaek, Denmark) at 55 °C overnight, followed by incubation with antibody against digoxin (Roche) and staining. ISH signals for *LINC00589* expression were determined in the form of the mean optical density using the AxioVision Rel.4.6 computerized image analysis system. The staining index (SI) was determined based on both the intensity and proportion of *LINC00589*. The expression of *LINC00589* was evaluated using the SI and scored as 0, 1, 2, 3, 4, 6, or 9. *LINC00589* expression was defined as high (SI ≥ 4) or low (SI < 4) based on the distribution of the frequency of SI scores.

### Statistics analysis

Data were analyzed using SPSS 19.0 software for windows. The results are presented as the mean ± SD. Statistical analysis was performed using the Student’s *t* test or analysis of variance (ANOVA) to compare means of the two groups or multiple groups of in vitro and in vivo data. The *χ*^2^ test was used to compare percentages or the association between *LINC00589* and clinicopathological parameters. Multivariate Cox regression was used to analyze independent prognostic factors for overall survival in HER2-positive breast cancer patients. The Spearman correlation test was performed to identify the correlation between the mRNA expression of target genes. Receiver Operating Characteristic (ROC) curve analysis was conducted, and the cutoff value was used to discriminate trastuzumab-responding or non-responding HER2-positive breast cancer patients. All data graphs were drawn using the PRISM Software, Version 9 (GraphPad Software, CA, USA). A value of *P* < 0.05 was considered statistically significant.

## Supplementary information

Supplementary Material

## Data Availability

The microRNA microarray data of breast cancer tissues have been deposited in NCBI Gene Expression Omnibus database (GEO) and are openly available via accession https://identifiers.org/geo: GSE47011^1^. The data generated and analyzed during this study are described in the following data record: https://datadryad.org/stash/share/V-yClLvMfSb1VQKqCILYac5iZjDzxhaLCmDX7UEekVc, or contacted to QH Ji to access this data. For clinical research purposes, this data is anonymized. Files underlying the clinical data are openly available in Excel format. All other data supporting the study can be found in the supplementary information file, and the corresponding author can make any materials available upon request. Un-cropped gels and western blots for Figs. 1–7 are included in supplementary materials (Supplementary Fig. 11). Western blot quantifications are included in supplementary materials (Supplementary Figs. 12–16). Graphically account for all FACS sequential gating are included in supplementary materials (Supplementary Fig. 17).
