# Supplementary material for: LINC00589-dominated ceRNA networks regulate multiple chemoresistance and cancer stem cell-like properties in HER2+ breast cancer
Source: NPJ Breast Cancer. 2022 Oct 29;8:115. doi: 10.1038/s41523-022-00484-0 (PMC9617889; doi:10.1038/s41523-022-00484-0)
Supplement: Supplementary file 1 — Supplementary Material [file 41523_2022_484_MOESM1_ESM.docx]

# Supplementary figures


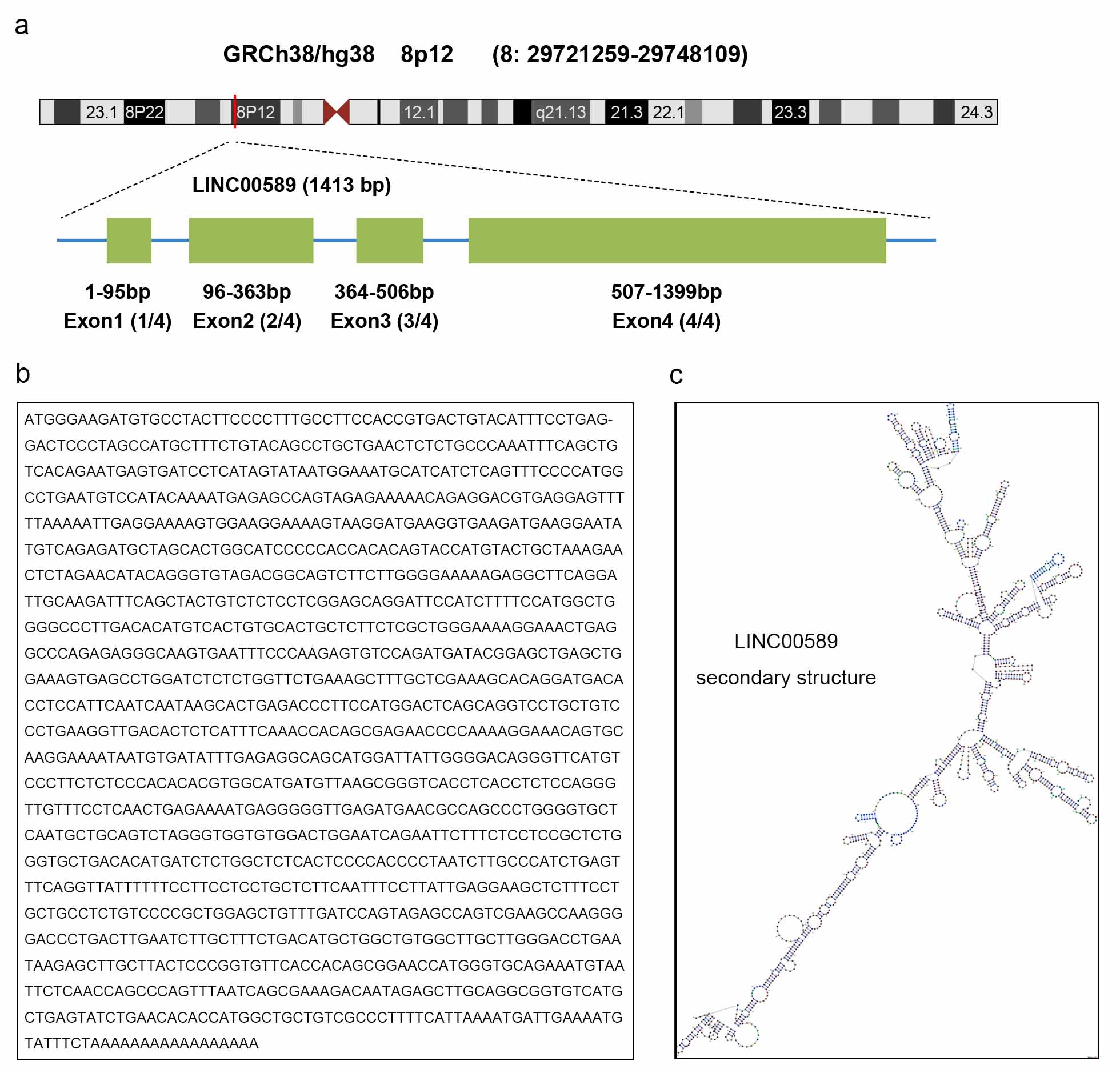


**Supplementary Figure 1. The localization, sequence and secondary structure of *LINC00589*. a** The genomic locus of *LINC00589* in humans and its exons are shown as a schematic diagram. **b** The sequence of *LINC00589*. **c** The secondary structure of *LINC00589* was predicted by AnnoLnc (http://annolnc.cbi.pku.edu.cn/).


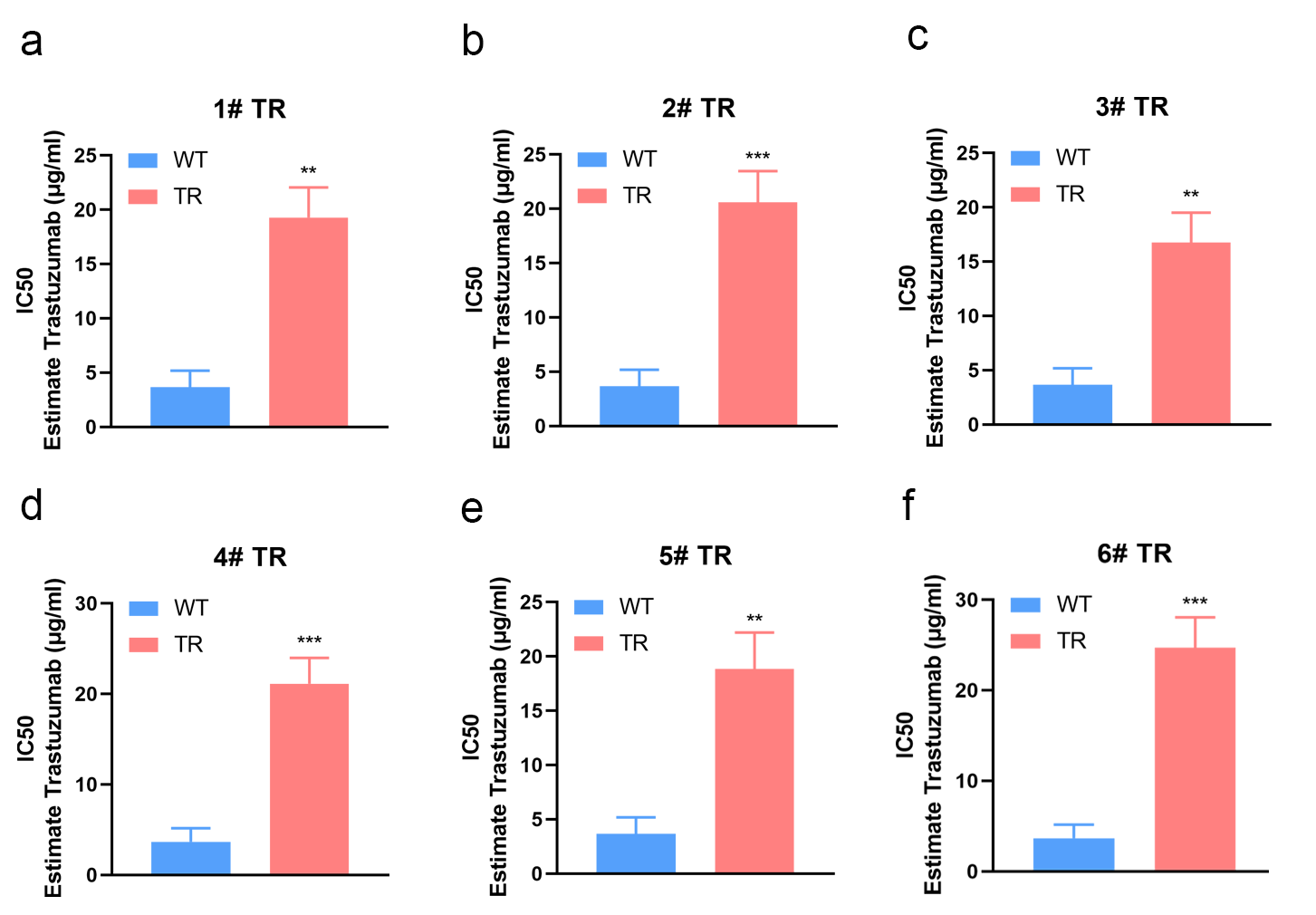


**Supplementary Figure 2. The IC50 of 5 trastuzumab-resistant (TR) clones.** **a-f** The IC50 represents the dose of 5 trastuzumab-resistant (TR) clones as measured by cell viability CCK-8 assay. Data are shown as mean ± SD; two-tailed *t* test was used to analyze the data in (a to f). ***P*<0.01 and ****P*<0.001. All data are representative of three independent experiments.


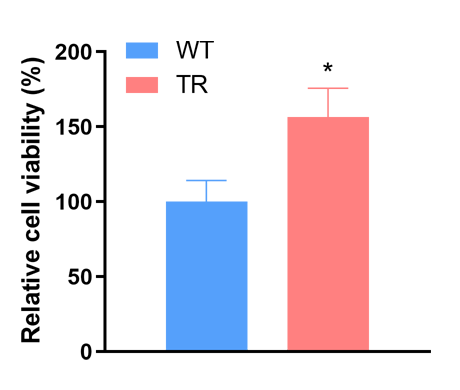


**Supplementary Figure 3. Validation of a trastuzumab resistant cell model.** WT and TR cells were treated with 5 μg/ml trastuzumab for 48 h, and the cell viability was determined by CCK-8 assay. Data are shown as mean ± SD; two-tailed *t* test was used to analyze the data. **P*<0.05. All data are representative of three independent experiments.


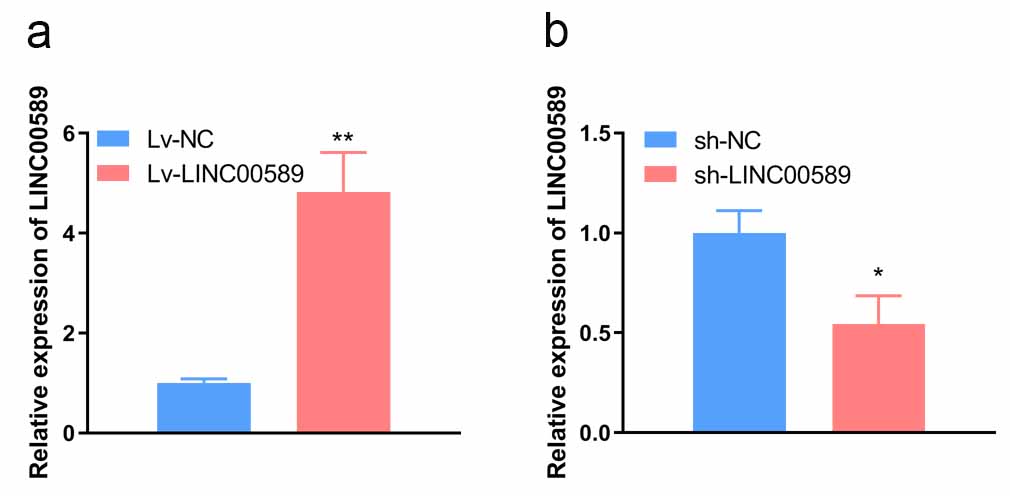


**Supplementary Figure 4. Validation of efficacy for *LINC00589* overexpression or knockdown lentiviruses in breast cancer cells.** **a, b** TR breast cancer cells were infected with the NC, *LINC00589* overexpression or knockdown lentiviruses, and *LINC00589* expression was determined by qRT-PCR assay. *GAPDH* was used as the internal control. Data are shown as mean ± SD; two-tailed *t* test was used to analyze the data in (a and b). **P*<0.05 and ***P*<0.01 versus negative control (NC). All data are representative of three independent experiments.


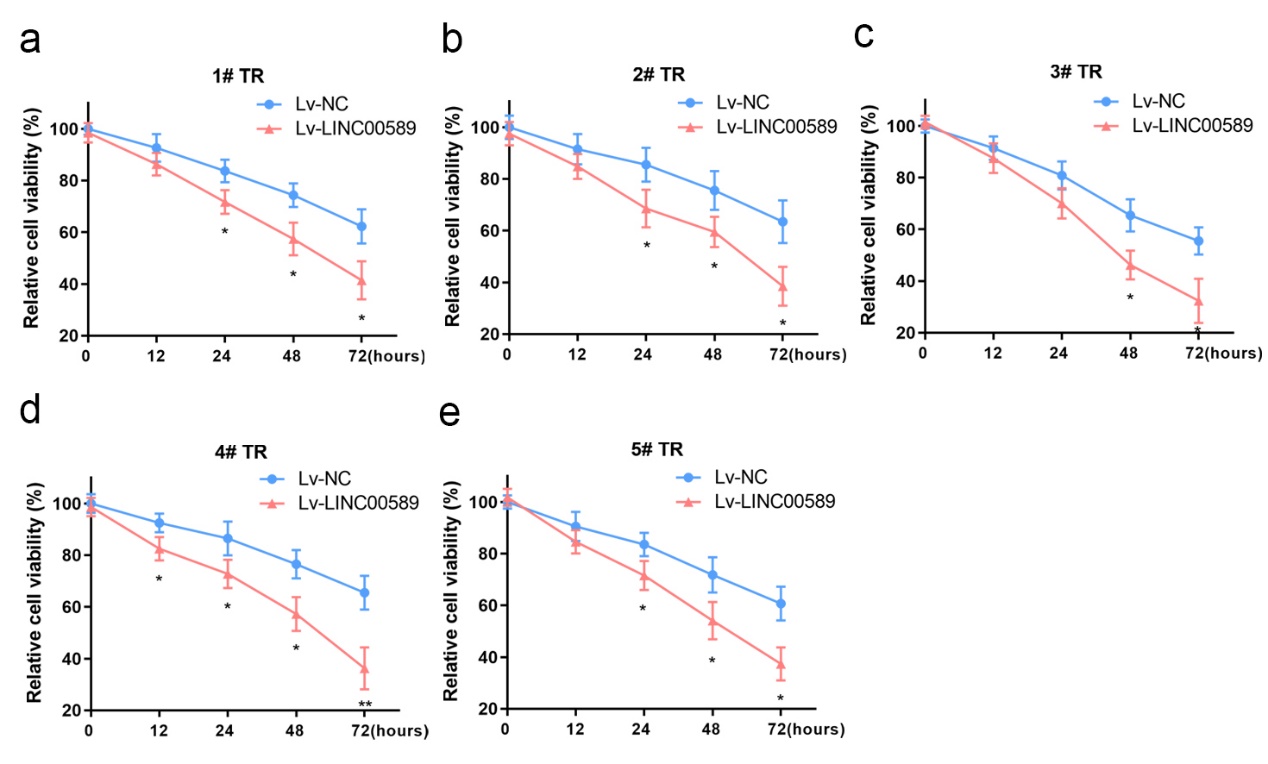


**Supplementary Figure 5. *LINC00589* promotes sensitivity of breast cancer cells to trastuzumab.** **a-e** 1#-5# TR SKBR3 clone cells were infected with NC or *LINC00589*-overexpression lentivirus and treated with 25 μg/ml trastuzumab. Data are shown as mean ± SD; two-way ANOVA was used to analyze the data in (a and b). **P*<0.05 and ***P*<0.01. All data are representative of three independent experiments.


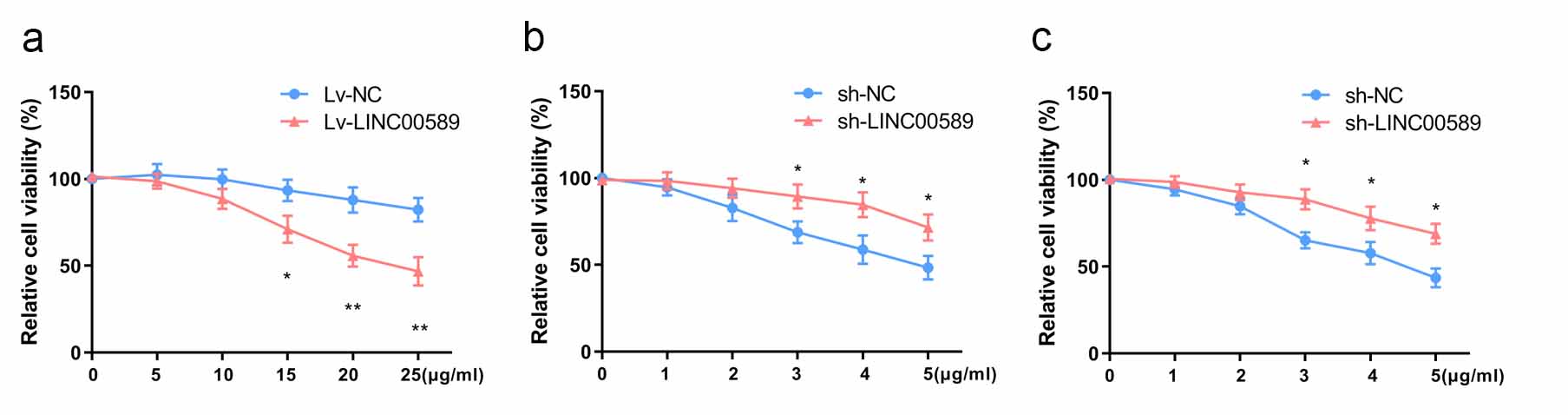


**Supplementary Figure 6. *LINC00589* regulates the sensitivity of TR and WT breast cancer cells to different concentrations of trastuzumab. a** TR SKBR3 cells were infected with NC or *LINC00589* lentiviruses and then treated with increasing concentrations of trastuzumab for 48 h. The cell viability was determined by CCK-8 assay. **b, c** WT SKBR3 cells and BT474 cells were infected with the sh-NC and sh-*LINC00589* lentiviruses for 48 h and treated using different concentration of trastuzumab. Then the cell viability was examined by CCK-8 assay. Data are shown as mean ± SD; two-way ANOVA was used to analyze the data in (a to c). **P*<0.05, ***P*<0.01. All data are representative of three independent experiments. NC, Negative control.


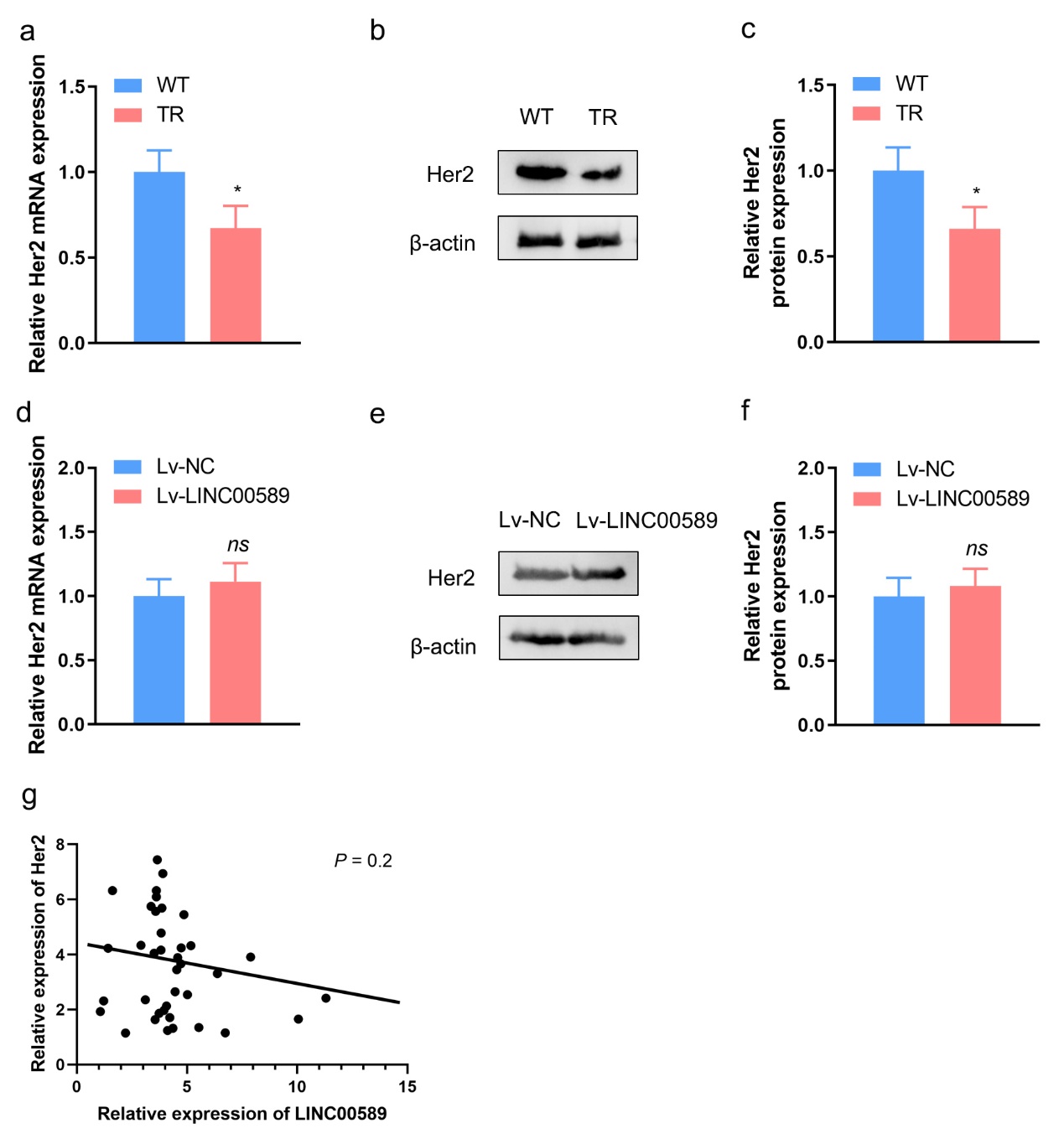


**Supplementary Figure 7. *HER2* expression in TR cells and *LINC00589* transfectants.** **a-c** *HER2* mRNA (a) and protein expression (b and c) in wide type (WT) and TR cells were tested by qRT-PCR and western blot assays. (c) Western blot quantification analysis of **b**. **d-f** Lv-NC and Lv-*LINC00589* were transfected in to the TR cells for 72 hours. *HER2* mRNA (d) and protein expression (d and f) in WT and TR cells were tested by qRT-PCR and western blot assays. (f) Western blot quantification analysis of **e**. *GAPDH* was used as the internal control for qRT-PCR and *β-actin* was used as an internal loading control for western blotting. Two-tailed *t* test was used to analyze the data in (a, c, d and f). **P*<0.05 versus negative control (NC). n.s., not significant. **g** Correlation between *LINC00589* and *HER2* expression in HER2-positive breast cancer patients’ tissues. *LINC00589* and *HER2* expression were determined by qRT-PCR and the correlation between them was analyzed by the Spearman correlation test. Data are shown as mean ± SD; **P*<0.05 versus negative control (NC). n.s., not significant.

#
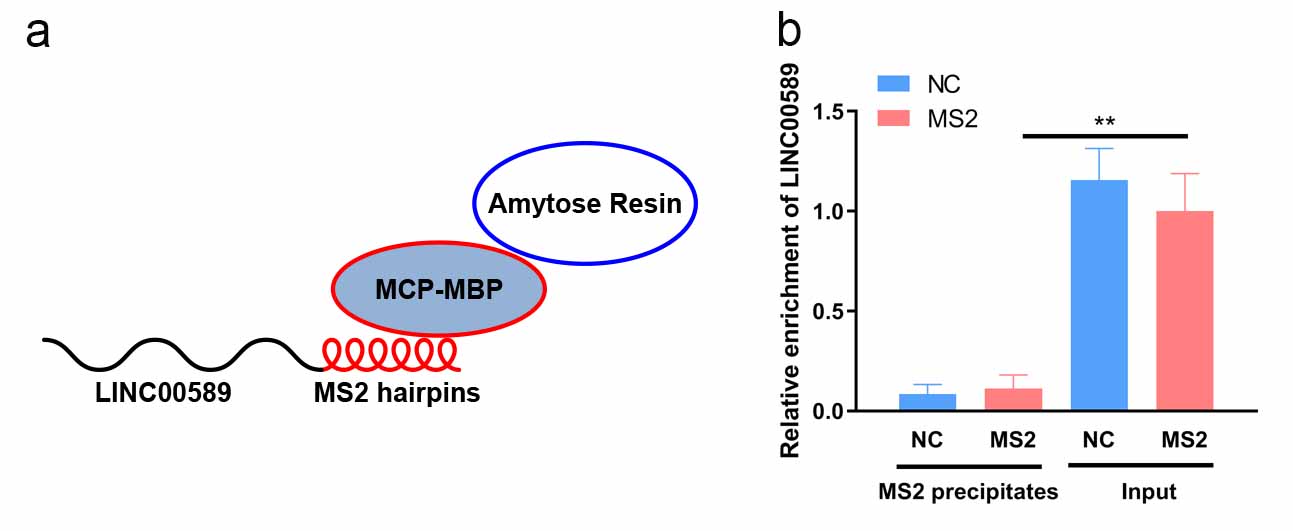


# Supplementary Figure 8. Validation of MS2 pull down system in breast cancer cells. a Schematic diagram of MS2 pull down assay for *LINC00589*. b WT cells were transfected with the control and MS2-*LINC00589* vector for 48 h, and the precipitated RNA was examined by qRT-PCR assay. Data are shown as mean ± SD; two-tailed *t* test was used to analyze the data in (b). ***P*<0.01. All data are representative of three independent experiments.


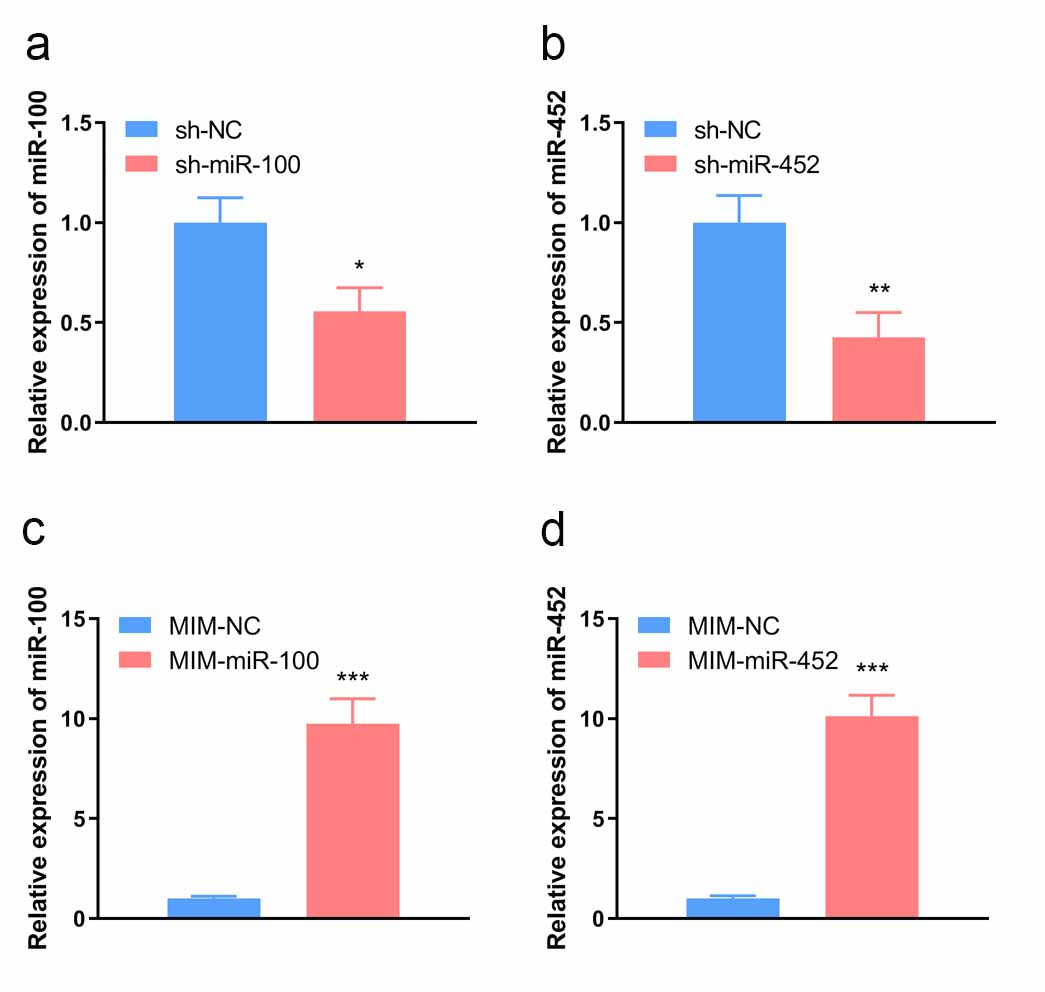


**Supplementary Figure 9. Validation of knockdown efficacy for miRNA in breast cancer cells.** **a, b** TR breast cancer cells were transfected with lentiviruses expressing shRNAs for *miR-100* and *miR-452*, and *miR-100* **(a)** and *miR-452* **(b)** expression were determined by qRT-PCR assay. **c, d** TR cells were transfected with the MIMIC control (MIM-NC), *miR-100* mimics or *miR-452* mimics, and *miR-100* **(c)** and *miR-452* **(d)** expression were tested by qRT-PCR assay. *U6* was used as the internal control. Data are shown as mean ± SD; two-tailed *t* test was used to analyze the data in (a to d). **P*<0.05, ***P*<0.01 and ****P*<0.001 versus negative control (NC). All data are representative of three independent experiments.


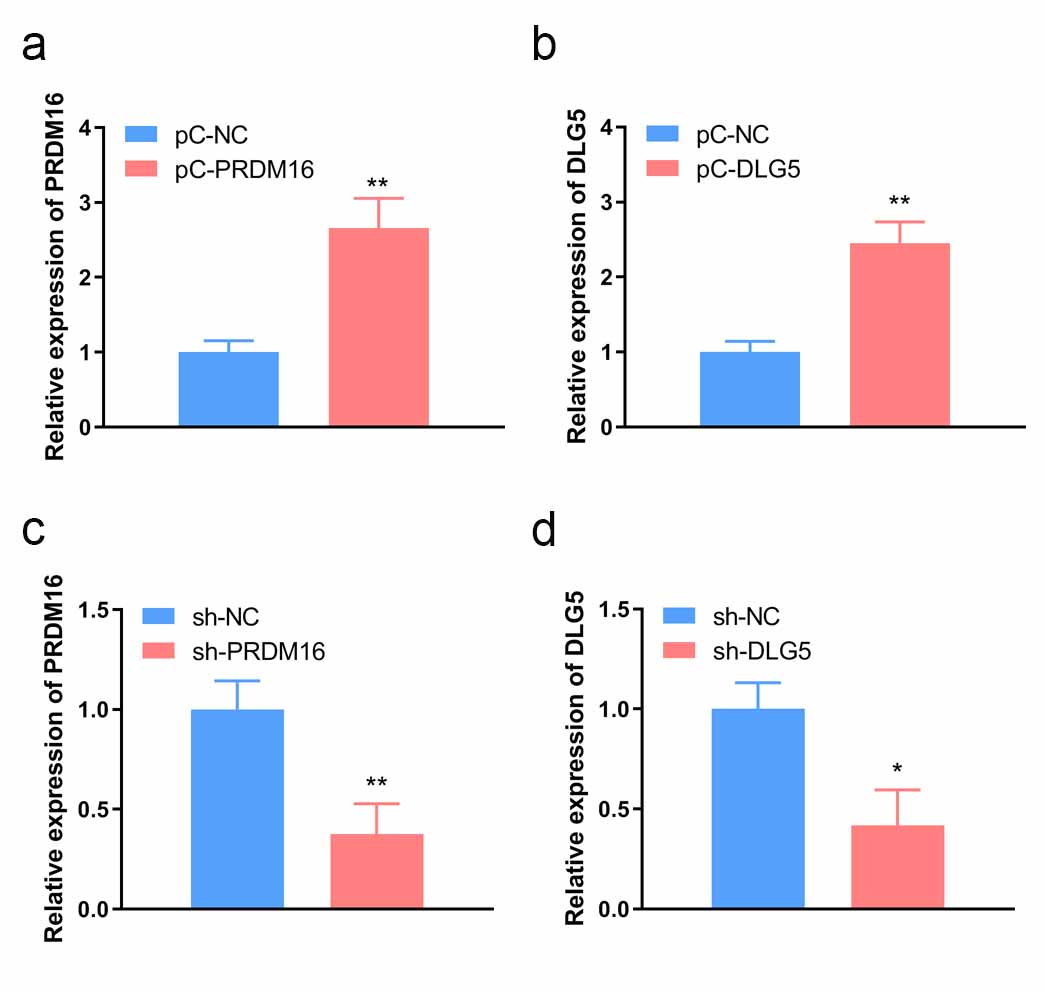


**Supplementary Figure 10. Validation of knockdown and overexpression efficacy for gene in breast cancer cells.** **a, b** TR cells were transfected with the control vector, pCDNA-*PRDM16* vector or pCDNA-*DLG5* vector, *PRDM16* **(a)** and *DLG5* **(b)** expression were tested by qRT-PCR assay. *GAPDH* was used as the internal control. **c, d** TR breast cancer cells were transfected with the sh-NC, sh-*PRDM16* and sh-*DLG5*, and *PRDM16* **(c)** and *DLG5* **(d)** expression were determined by qRT-PCR assay. Data are shown as mean ± SD; two-tailed *t* test was used to analyze the data in (a to d). **P*<0.05 and ***P*<0.01 versus negative control (NC). All data are representative of three independent experiments.

**Supplementary Figure 11. Uncropped blots**

**For Figure 3e**


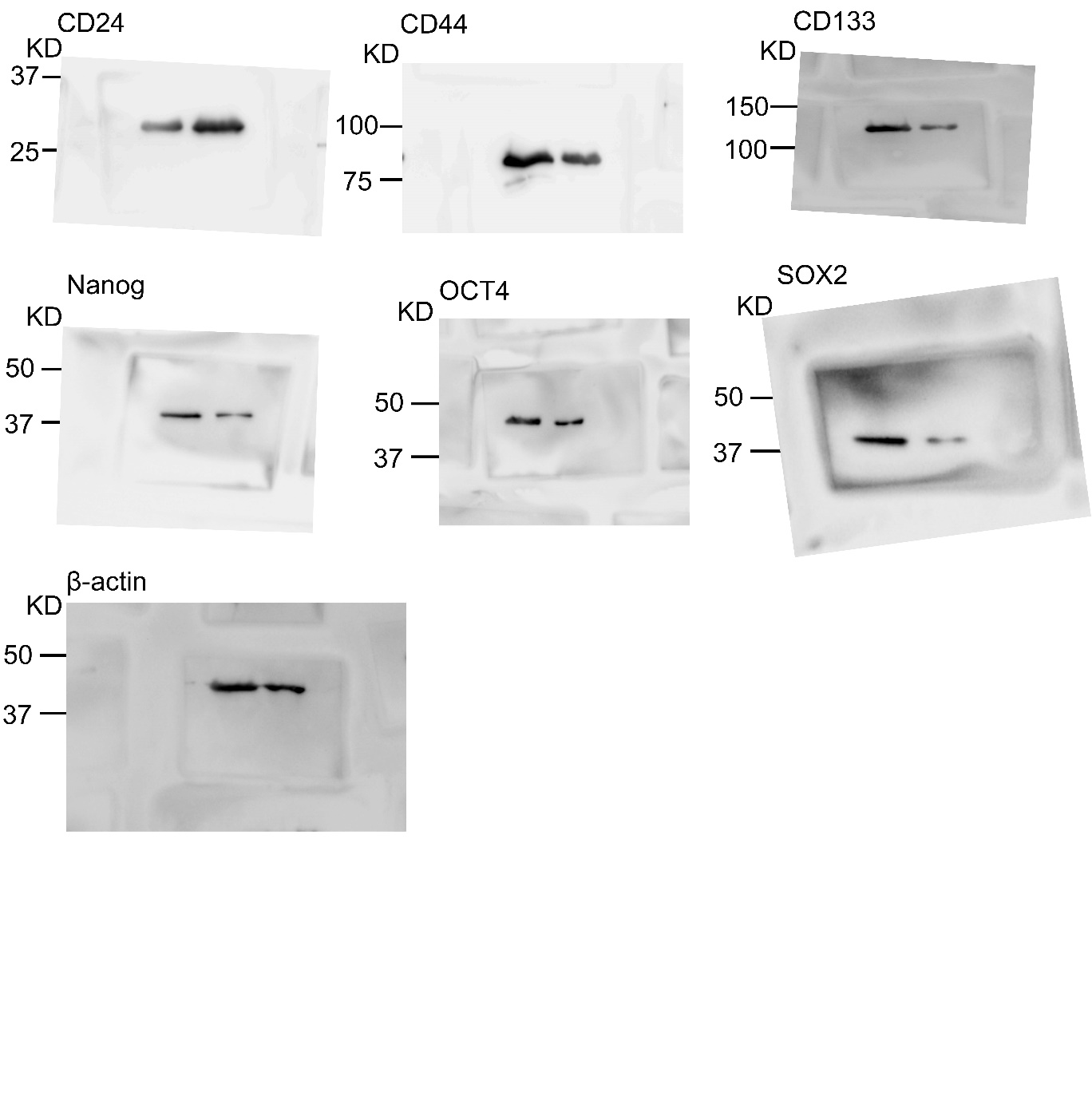


**For Figure 5l**


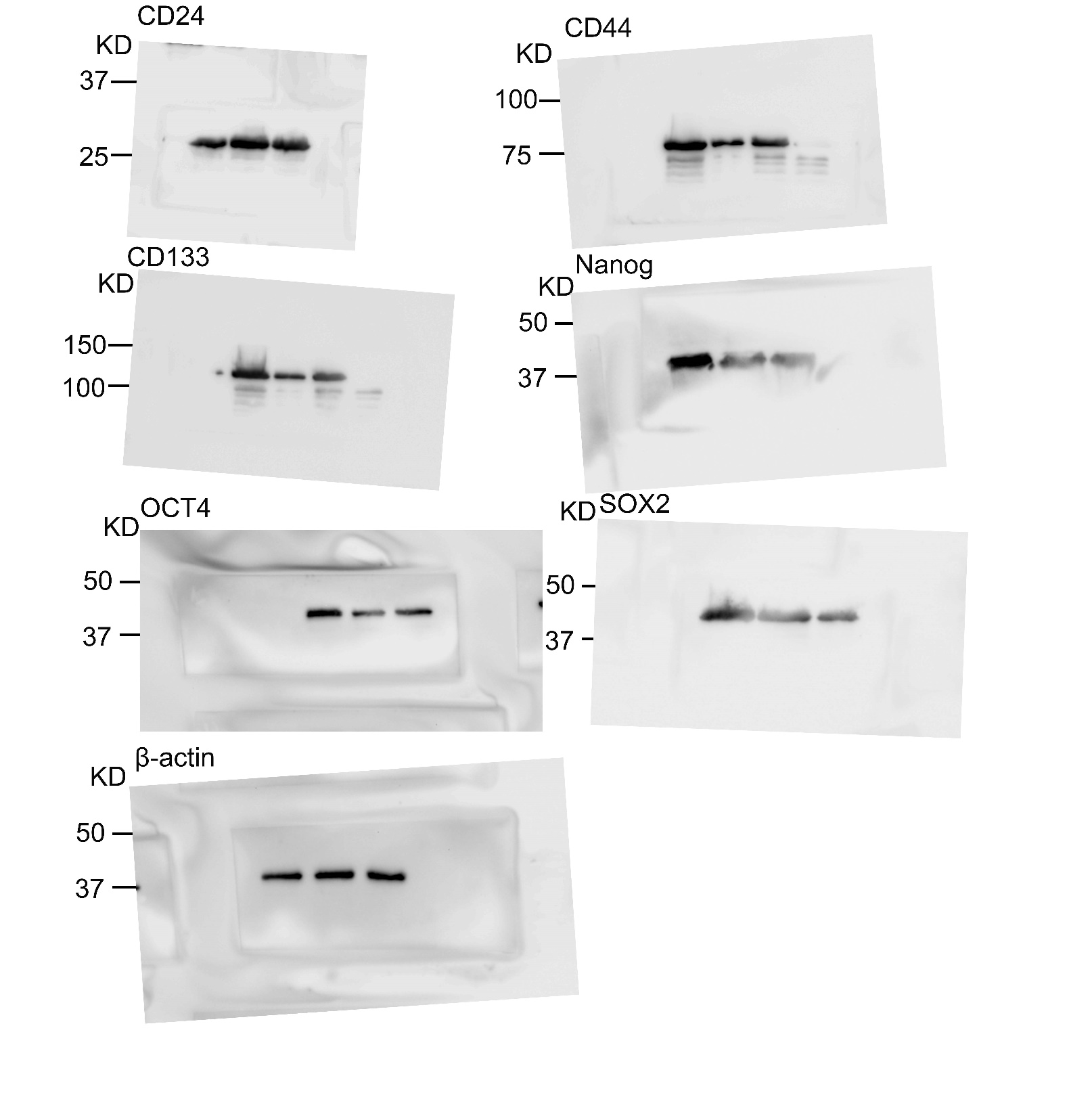


**For Figure 6e, 6g and 6i**


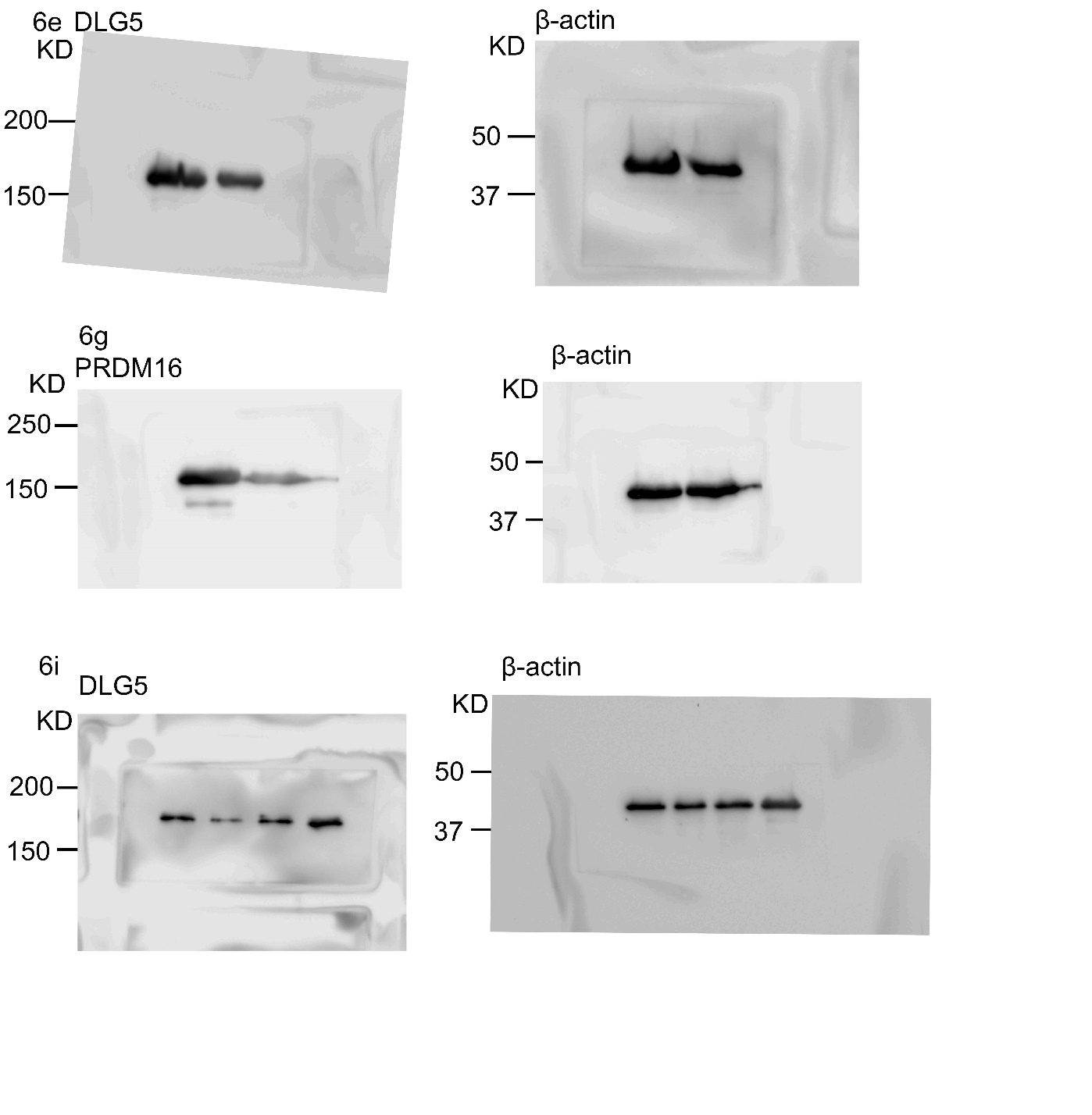


**For Figure 6k and 6m**


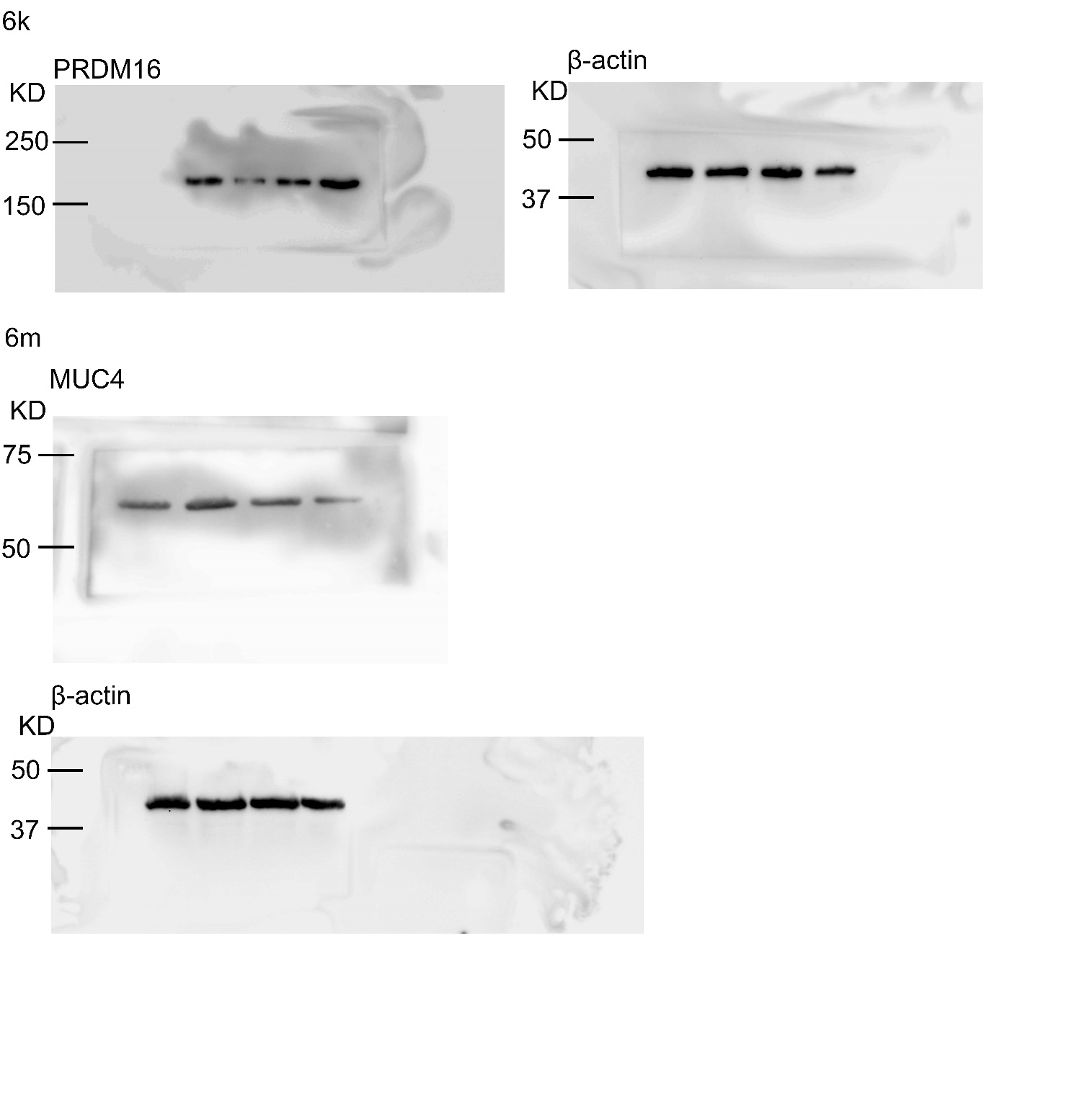


**For Figure 7l**


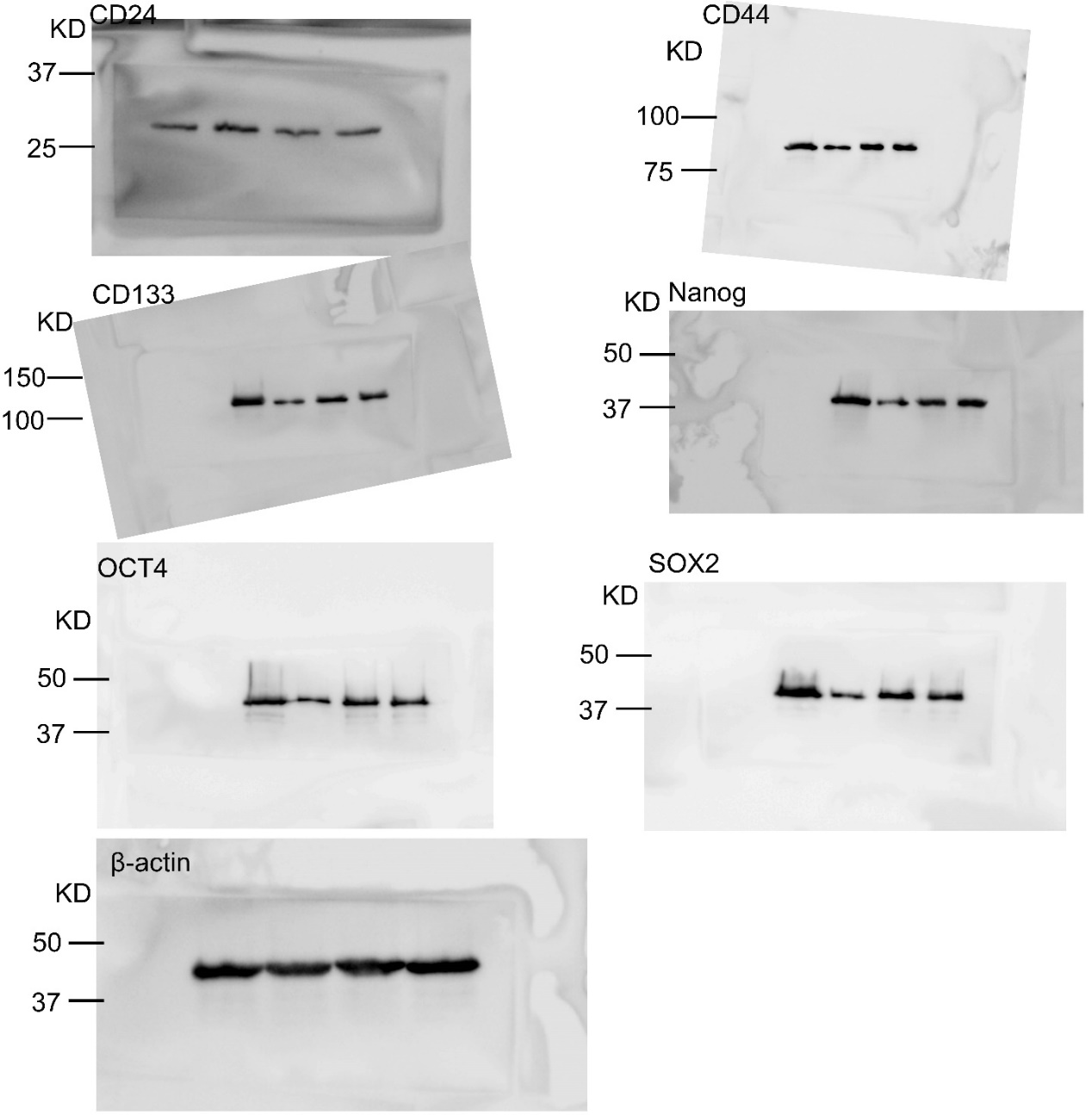


**Supplementary Figure 12.**


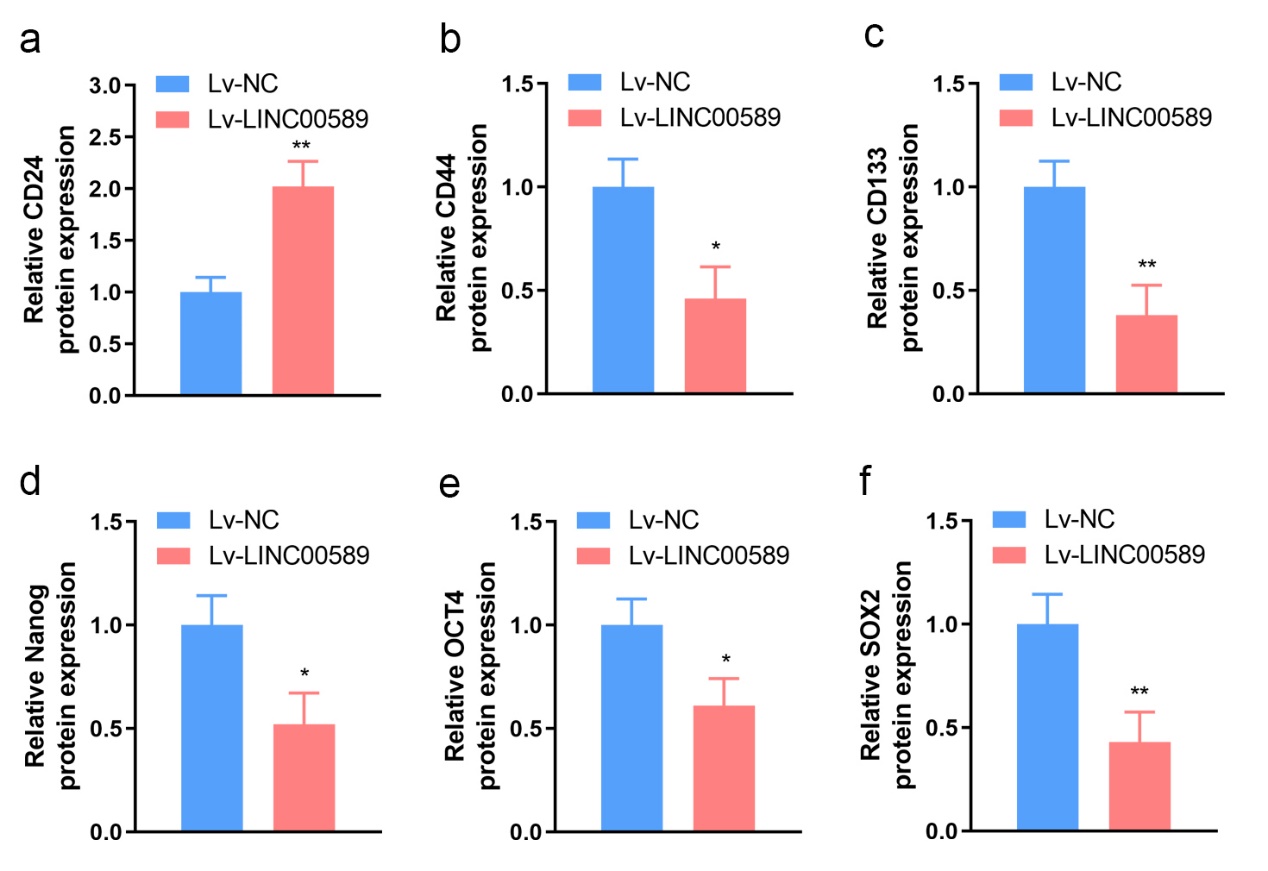


**Supplementary Figure 12. Western blot quantification analysis of figure 3e.** Bar plots are depicted relative to β-actin protein levels, arbitrarily set to 1. CD24 (a), CD44 (b), CD133 (c), Nanog (d), OCT4 (e) and SOX2 (f) of blots represents data obtained from a single gel and experiment. Results shown are representative of 3 independent experiments. Data are represented as the mean ± SD; two-tailed *t* test was used to analyze the data in (a to f). **P*<0.05, ***P*<0.01.

**Supplementary Figure 13.**


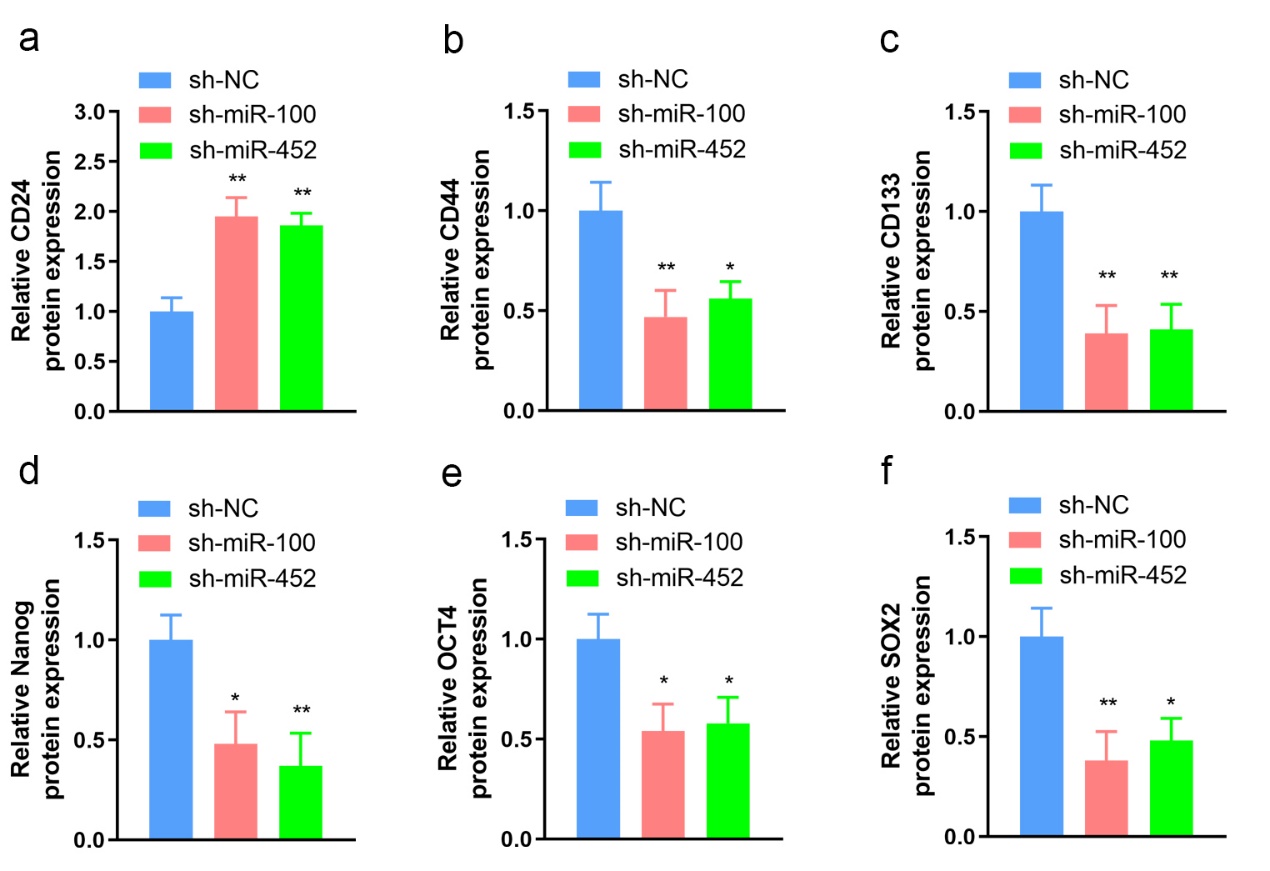


**Supplementary Figure 13. Western blot quantification analysis of figure 5l.** Bar plots are depicted relative to β-actin protein levels, arbitrarily set to 1. CD24 (a), CD44 (b), CD133 (c), Nanog (d), OCT4 (e) and SOX2 (f) of blots represents data obtained from a single gel and experiment. Results shown are representative of 3 independent experiments. Data are represented as the mean ± SD; two-tailed *t* test was used to analyze the data in (a to f). **P*<0.05, ***P*<0.01.

**Supplementary Figure 14.**


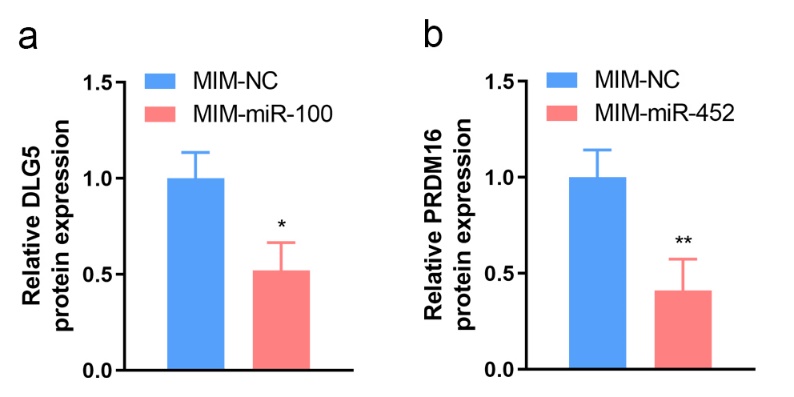


**Supplementary Figure 14. Western blot quantification analysis of figure 6e and 6g.** Bar plots are depicted relative to β-actin protein levels, arbitrarily set to 1. DLG5 (a) and PRDM16 (b) of blots represents data obtained from a single gel and experiment. Results shown are representative of 3 independent experiments. Data are represented as the mean ± SD; two-tailed *t* test was used to analyze the data in (a and b). **P*<0.05, ***P*<0.01.

**Supplementary Figure 15.**


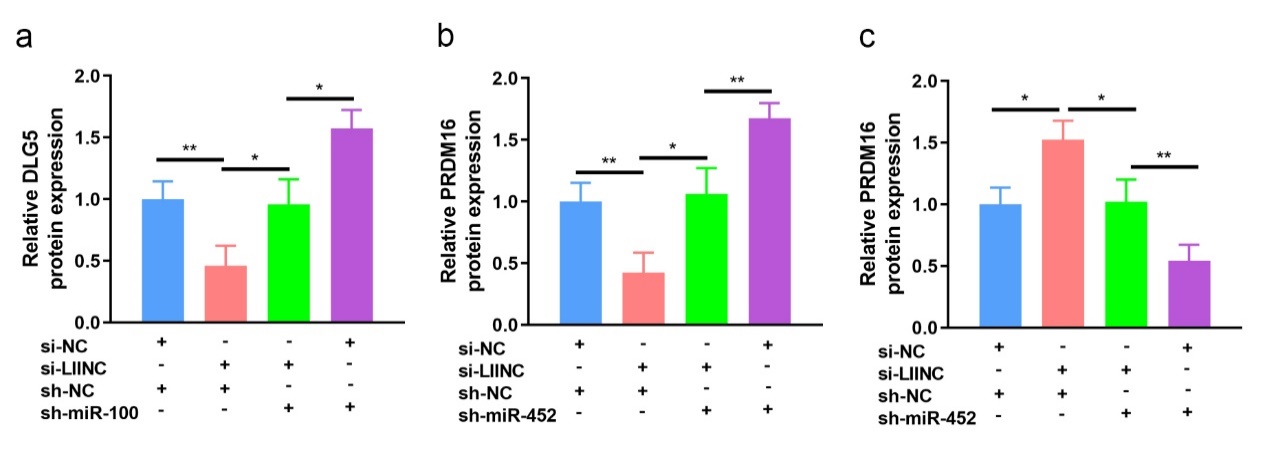


**Supplementary Figure 15. Western blot quantification analysis of figure 6i, 6k and 6m.** Bar plots are depicted relative to β-actin protein levels, arbitrarily set to 1. DLG5 (a), PRDM16 (b) and MUC4 (c) of blots represents data obtained from a single gel and experiment. Results shown are representative of 3 independent experiments. Data are represented as the mean ± SD; two-tailed *t* test was used to analyze the data in (a to c). **P*<0.05, ***P*<0.01.

**Supplementary Figure 16.**


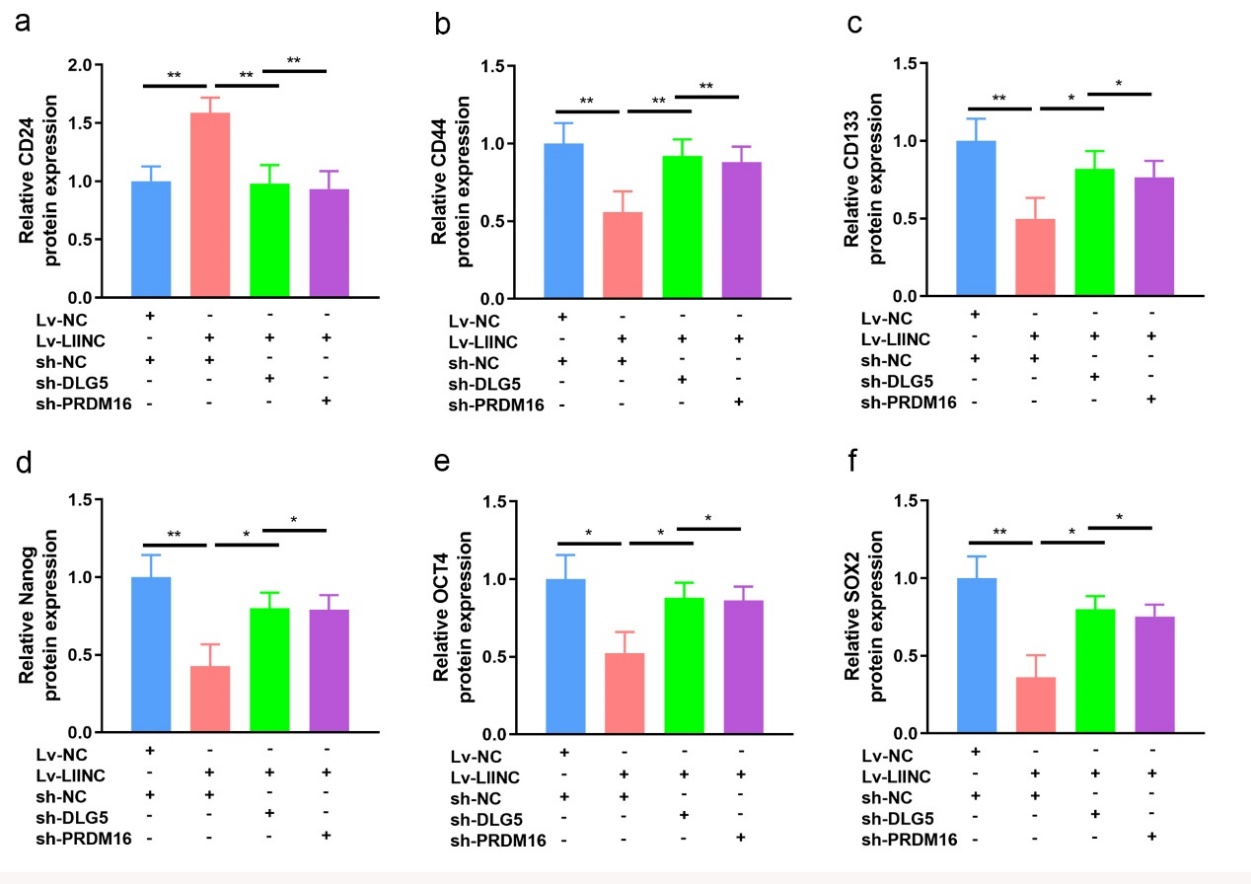


**Supplementary Figure 16. Western blot quantification analysis of figure 7l.** Bar plots are depicted relative to β-actin protein levels, arbitrarily set to 1. CD24 (a), CD44 (b), CD133 (c), Nanog (d), OCT4 (e) and SOX2 (f) of blots represents data obtained from a single gel and experiment. Results shown are representative of 3 independent experiments. Data are represented as the mean ± SD; two-tailed *t* test was used to analyze the data in (a to f). **P*<0.05, ***P*<0.01.

**Supplementary Figure 17.**


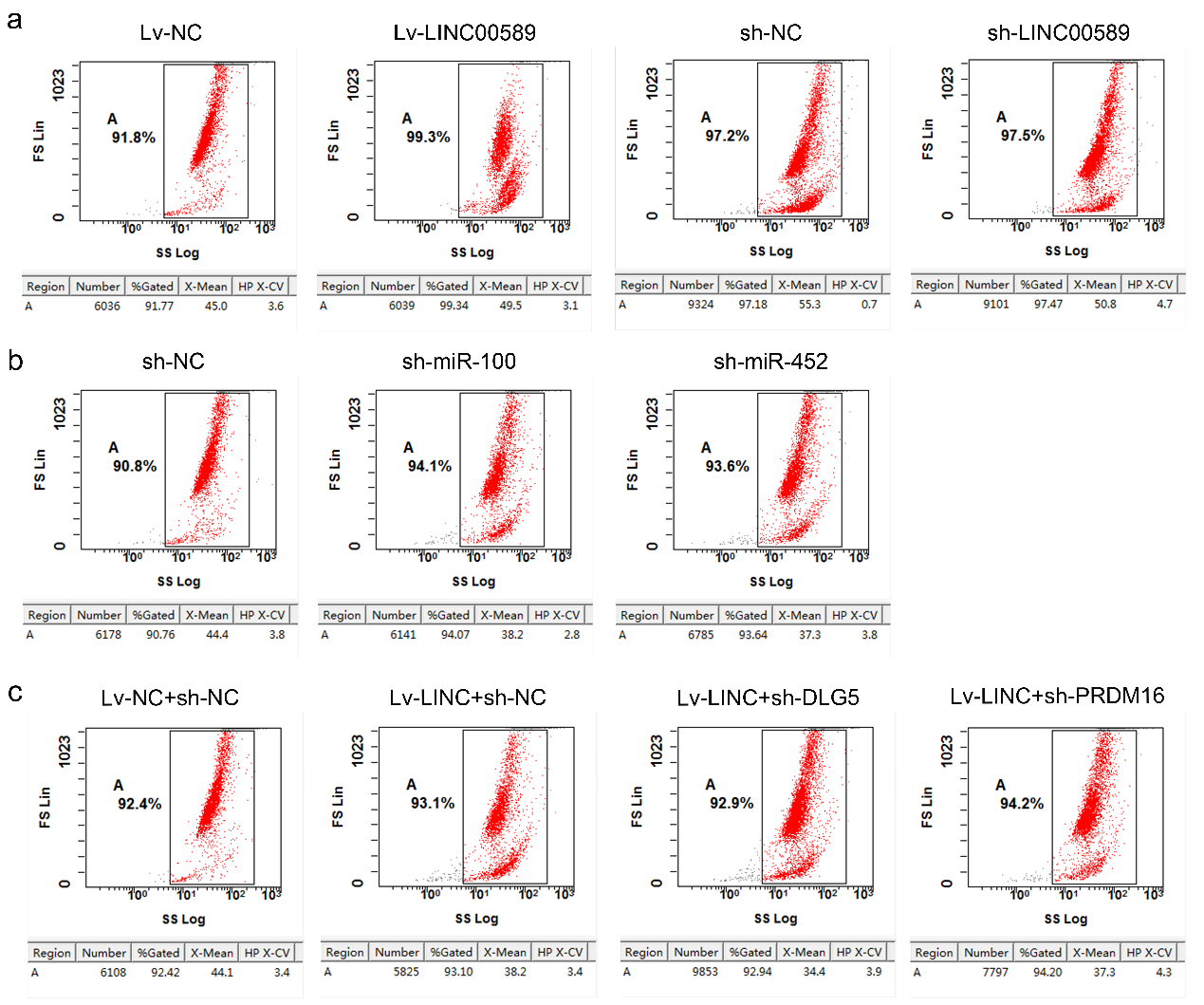


**Supplementary Figure 17.** **Graphically account for all FACS sequential gating.** FACS sequential gating for Figure 2d (a), Figure 5d (b) and Figure 7d (c).
